# Toward a third term of Health Japan 21 – implications from the rise in non-communicable disease burden and highly preventable risk factors

**DOI:** 10.1016/j.lanwpc.2021.100377

**Published:** 2022-01-23

**Authors:** Shuhei Nomura, Haruka Sakamoto, Cyrus Ghaznavi, Manami Inoue

**Affiliations:** a Department of Global Health Policy, Graduate School of Medicine, The University of Tokyo, Tokyo, Japan; b Department of Health Policy and Management, School of Medicine, Keio University, Tokyo, Japan; c Tokyo Foundation for Policy Research, Tokyo, Japan; d Division of Prevention, National Cancer Center Institute for Cancer Control, Tokyo, Japan

**Keywords:** Health Japan 21, Health crisis, Non-communicable diseases, Preventable risk factors

## Abstract

In 2000, the Japanese government launched the National Health Promotion Movement known as Health Japan 21 (HJ21), a 13-year national health promotion policy (2000–2012) aimed at preventing and controlling non-communicable diseases (NCDs) and their underlying risk factors. After the revision in 2013 (2013–2023), the target NCDs and risk factors are being reviewed and a new strategy for the third term of HJ21 is going to be discussed. Using the latest findings from the Global Burden of Disease Study 2019, this paper highlights NCDs that continue to increase health losses and preventable metabolic and behavioural risk factors. These NCDs and risk factors are associated with an increased risk of serious illness and death from the novel coronavirus disease (COVID-19). The third term of HJ21 will be formulated during the continuing threat of acute health crises like the current COVID-19 pandemic and thus offers an important opportunity to renew public health efforts to halt the growing burden of NCDs in Japan. This article may serve as one of the roadmaps for the formulation of the third term of HJ21.

## Introduction

In recent years, Japan's disease structure has been changing due to the rapid aging of the population and lifestyle changes.^1^ As a result, there are ever-increasing deaths and disabilities associated with non-communicable diseases (NCDs), such as cancer, cardiovascular diseases, and diabetes.^1^ These NCDs have accounted for more than 30% of national healthcare expenditures.^2^ In 2000, the Japanese government launched the National Health Promotion Movement in the 21st Century, known as Health Japan 21 (HJ21), a new 13-year national health promotion policy (2000–2012) aiming to prevent and control lifestyle-related NCDs and their underlying risk factors.^3^^,^^4^ Seventy-nine targets were set in nine areas (nutrition and diet, physical activity and exercise, promotion of rest and mental health, smoking, alcohol use, dental and oral health, diabetes, cardiovascular disease, and cancer), of which only 17% were achieved, primarily in the area of dental and oral health.^5^ The second term of HJ21 runs from 2013 to 2023 (originally a 10-year plan through 2022, but later revised and extended to 2023) with a greater focus on extending healthy life expectancy and reducing health inequality, while maintaining the same scope to address NCDs. We have been involved in the generation of evidence to inform the formulation and revision of HJ21 and the design of the basic strategy for its implementation. The final evaluation of the second term of HJ21 began in mid-2021 and the third term of HJ21 is scheduled to start in 2024.

Using the latest findings from the Global Burden of Disease Study 2019 (GBD 2019),^6^ this article sought to: (1) provide an overview of NCDs and their risk factors in Japan from the perspective of disability-adjusted life years (DALYs) and deaths; (2) review how the NCDs and risk factors highlighted in the GBD 2019 have been addressed in the second term of HJ21 and summarize the HJ21 interim evaluation; (3) discuss the need for further public health efforts to stem the rise of NCDs and preventable risk factors. The third term of HJ21 will be formulated in the midst of the continuing threat of the coronavirus disease (COVID-19) pandemic and provides an important opportunity to renew public health efforts regarding NCDs in Japan. We hope that this article will serve as a roadmap for the formulation of the third term of HJ21 to assist efforts in Japan to ensure a healthier population, making the country more resilient to the threat of future pandemics.

## Methods

In this paper, we used the GBD 2019 data published by the Institute for Health Metrics and Evaluation (IHME).^6^ GBD is one of the largest systematic scientific efforts in the world, providing a data-rich framework for comparing the importance of different diseases, injuries, and risk factors that cause premature death and disability over time. All estimated data are openly available, and the value of GBD lies not only in the data, but also in the critical discussions made possible by its comprehensiveness and longitudinal nature.^7^ GBD allows for a clearer and more rigorous discussion that should guide better population health planning.

We considered the estimates of healthy life expectancy in 2013 and 2019 as well as DALYs and deaths in these years for 123 underlying NCDs cause groups (including injuries) and 29 behavioural and metabolic risks as preventable risk factors. Healthy life expectancy provides a summary metric of the number of years one can live a healthy life, while DALYs is a summary metric of the number of healthy years lost (measured as the sum of years of life lost [YLLs] and years lived with disability [YLDs]) at the population level.^8^ A detailed description of general methodological approaches of GBD 2019 has been described elsewhere.^9^, ^10^, ^11^ Unless otherwise noted, all data on the burden of disease in this article were extracted from the GBD 2019 through the freely available platform^12^ and the estimates of DALYs and deaths presented in this paper refer to both sexes combined at all ages and do not include burden of disease associated with communicable, maternal, neonatal, and nutritional diseases.

In addition to the 29 preventable behavioural and metabolic risk factors addressed in the GBD 2019, we considered the following five infections, which are known to cause a large burden of disease in cancer patients in Japan,^13^, ^14^, ^15^ as important and preventable cancer risk factors: hepatitis B virus, hepatitis C virus, *Helicobacter pylori*, human papillomavirus (HPV), and human T-cell leukemia virus type 1 (HTLV-1). Since the GBD 2019 estimated DALYs and deaths from cancer caused by hepatitis B and C, we treated those values as the cancer disease burden attributable to hepatitis B and C virus infection as risk factors. For *Helicobacter pylori* infection, HPV infection, and HTLV-1 infection, we considered only cancer deaths using the following method. First, the population attributable fractions (PAFs) of *Helicobacter pylori* infection in total cancer deaths are estimated to be 9.4% for women and 12.4% for men according to previous research.^15^ Therefore, we calculated the number of cancer deaths attributable to *Helicobacter pylori* infection by multiplying the total number of cancer deaths by the PAFs. Additionally, the PAFs of HPV infection in deaths from various types of cancer (defined by ICD-10: the 10th revision of the International Statistical Classification of Diseases and Related Health Problems) were extracted from previous research (Supplementary Table 1).^15^^,^^16^ We then calculated the number of cancer deaths attributable to HPV infection by multiplying the number of deaths due to HPV-associated cancers by the corresponding PAF. Finally, for deaths attributable to HTLV-1 infection, the C91.5 code of ICD-10 was used. For the total number of cancer deaths, we used GBD 2019 estimates; for cancer deaths stratified by ICD-10 codes, we used vital statistical data, which was specifically prepared by the Vital, Health, and Social Statistics Office under the Ministry of Health, Labour, and Welfare (MHLW) and provided by the Health Service Division, Health Service Bureau, MHLW, based on Article 32 of the Statistics Act. These vital statistical data are presented in Supplementary Table 1. For the calculation of DALYs and deaths per 100,000 population (i.e., DALYs rate and mortality rate), the population size obtained from the GBD 2010 population estimates for Japan was used.^17^

## Search strategy and selection criteria

To inform this article, we conducted a PubMed search for peer-reviewed literature using the key words (“noncommunicable diseases” [MeSH Terms] and “risk factors” [MeSH Terms]). All relevant results until 25 October 2021 were considered with no language restrictions. Other relevant articles were identified via the snowballing method. Source types were limited primarily to academic publications, but gray literature sources, including from government agencies and relevant stakeholder organizations, were also included where relevant.

## Progressive NCDs and stagnation in health progress in Japan

The healthy life expectancy at birth in Japan has steadily increased by 0.74 years from 73.10 years in 2013 to 73.84 years in 2019, while life expectancy at birth has increased by 0.94 years from 83.86 years to 84.80 years. Thus, the number of years people survive with disability is increasing. The gap between prefectures with the lowest and highest healthy life expectancies has widened, from 1.80 to 2.13 years, and the standard deviation between prefectures also increased from 0.37 to 0.46 years in the same period. Disability as measured by YLDs, rather than premature death as measured by YLLs, has become an increasingly large share of DALYs in Japan—rising from 47.74% of total DALYs in 2013 to 48.23% in 2019.

**Figure 1:**
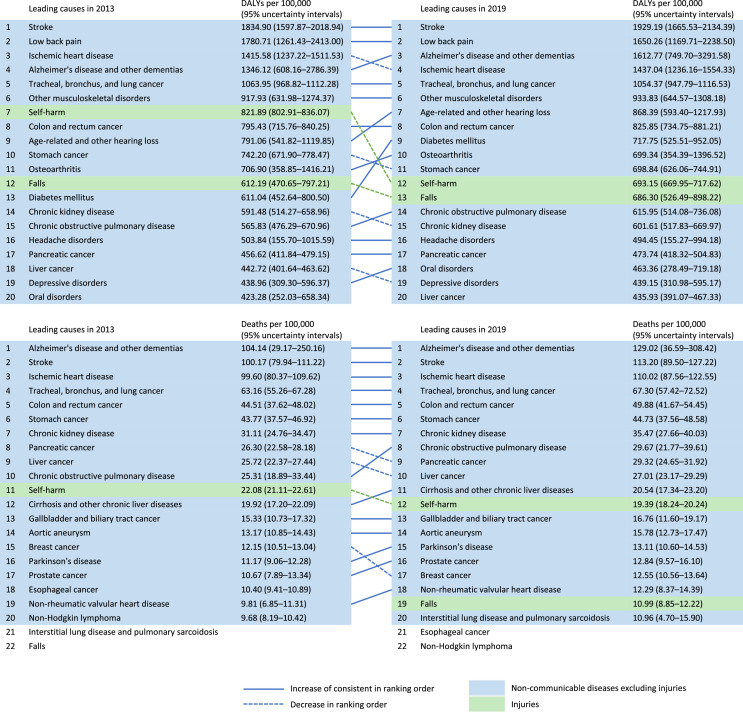
Causes of all-ages DALYs and deaths per 100,000 population in Japan in 2013 and 2019 for both sexes combined. * Data were obtained from the GBD results tool: http://ghdx.healthdata.org/gbd-results-tool.

More than 80% of all health losses, as measured by DALYs, are now due to NCDs. In 2019, the top 10 contributors to the current health loss from NCDs in DALYs in Japan include seven causes that primarily affect older adults: stroke (associated DALYs per population increased by 5.14% between 2013 and 2019), low back pain (decreased by 7.33%), Alzheimer's and other dementias (increased by 19.81%), ischemic heart disease (increased by 1.52%), lung cancer (decreased by 0.90%), age-related hearing loss (increased by 9.77%), and diabetes (increased by 17.46%) (Figure 1). Given the rapid increase seen for diabetes and Alzheimer's disease (Alzheimer's has also increased by 23.90% in deaths per population), these causes warrant close attention.

Furthermore, health gains are beginning to stagnate, with age-weighted health losses increasing by 1.33% between 2013 and 2019, as highlighted by past literature.^1^ Increases in ill-health could overburden a health system that is not adequately equipped to deal with the chronic diseases associated with an aging population.^18^^,^^19^ Although the relative ranking across causes for DALYs differs between women and men, a similar trend is observed in each (Supplementary Figures 1 and 2, respectively).

## Increase in risk factors contributing to NCDs

**Figure 2:**
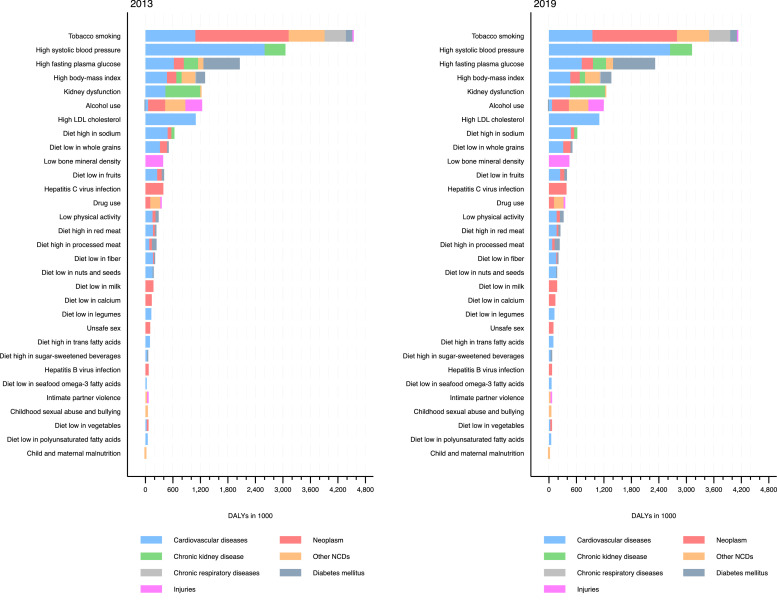
All-ages DALYs attributable to preventable behavioural and metabolic risk factors in 2013 and 2019 for both sexes combined. * Data, except for hepatitis B and C virus infections, were obtained from the GBD results tool: http://ghdx.healthdata.org/gbd-results-tool. DALYs: disability-adjusted life years; NCD: non-communicable disease; LDL: low-density lipoprotein. The order of risk factors is sorted by 2019 values.

There has been a particularly worrisome increase in exposure to several potentially preventable metabolic risks factors such as high systolic blood pressure, high fasting plasma glucose, high body-mass index (BMI), kidney dysfunction, high low-density lipoproteins (LDL) cholesterol, and low bone mineral density which has led to increased DALYs from NCDs (Figure 2), highlighting the urgent need for increased public health efforts to address these risk factors.^1^ Metabolic risk factors collectively accounted for 18.15% of total DALYs from NCDs in 2019, an increase since 2013 (17.36%). Potentially preventable behavioural risk factors, such as tobacco smoking, alcohol use, high-sodium diets, and diet low in whole grains are also notable issues (Figure 2). Behavioural risk factors, excluding hepatitis B and C virus infections, collectively accounted for 21.02% of total DALYs from NCDs in 2019, having decreased since 2013 (22.22%). These are among the top 10 risk factors for DALYs from NCDs in 2019 with smoking in first place, and little has changed in the rankings since 2013. DALYs per population from NCDs attributable to these risk factors have been rising since 2013, except tobacco smoking (Supplementary Figure 3). Precise data for the information presented in Figure 2 and Supplementary Figure 3 can be found in Supplementary Tables 2 and 3, respectively.

**Figure 3:**
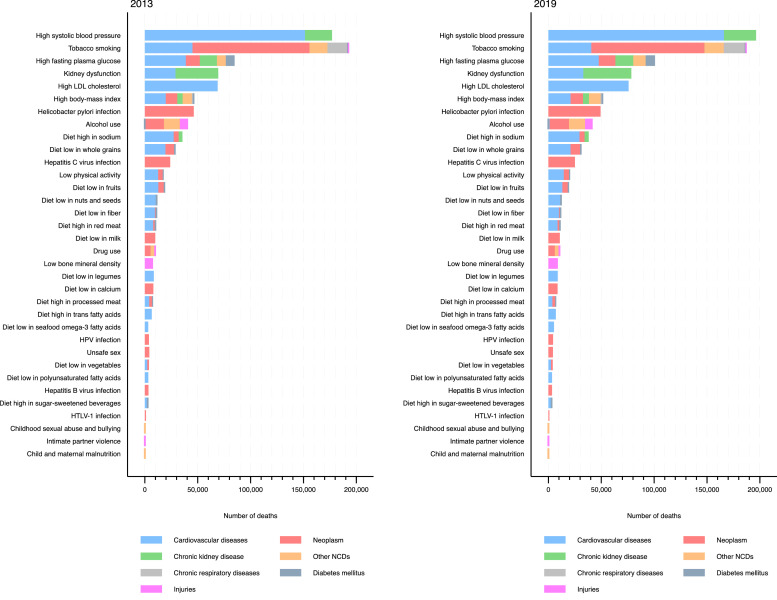
All-ages number of deaths attributable to preventable behavioural and metabolic risk factors in 2013 and 2019 for both sexes combined. * Data, except for hepatitis B and C virus infections, *Helicobacter pylori* infection, HPV infection, and HTLV-1 infection, were obtained from the GBD results tool: http://ghdx.healthdata.org/gbd-results-tool. NCD: non-communicable disease; LDL: low-density lipoprotein; HPV: human papillomavirus; HTLV-1: human T-cell leukemia virus type 1. The order of risk factors is sorted by 2019 values.

Metabolic risk is also contributing to a large number of deaths, with high systolic blood pressure accounting for 196,385 deaths in 2019 (Figure 3), followed by high fasting plasma glucose (causing 100,809 deaths), kidney dysfunction (78,418), high LDL cholesterol (75,782), and high BMI (51,822). In addition to these five metabolic factors, the top 10 risk factors for deaths from NCDs in 2019 included tobacco smoking (causing 187,238 deaths), *Helicobacter pylori* infection (49,284), alcohol use (41,554), high-sodium diets (38,087), and diets low in whole grains (30,877). Metabolic risk factors collectively accounted for 24.64% of the total number of deaths from NCDs in 2019, which has slightly increased since 2013 (24.22%). Additionally, behavioural risk factors, excluding hepatitis B and C virus infections, *Helicobacter pylori* infection, HPV infection, and HTLV-1 infection, collectively account for 25.96% of the total number of deaths from NCDs in 2019, which has decreased since 2013 (27.89%). Deaths per population from NCDs attributable to these risk factors have been rising since 2013, except tobacco smoking (Supplementary Figure 4). For hepatitis B virus infection, hepatitis C virus infection, *Helicobacter pylori*, HPV infection, and HTLV-1 infection, which we assessed in addition to the risk factors addressed in GBD 2019, the deaths per population increased for all but HTLV-1 infection from 2013 to 2019. Precise data for the information presented in Figure 3 and Supplementary Figure 4 can be found in Supplementary Tables 2 and 3, respectively. The major difference between women and men is that the relative ranking of tobacco smoking and alcohol use is much higher for men, in terms of both DALYs and deaths. Figures and data according to sex can be found in Supplementary Figures 5–12 and Supplementary Tables 4–7.

## Coverage of the second term of HJ21 and the interim evaluation of the progress in 2018

The second term of HJ21 contains 53 targets in its five chapters: (1) extension of healthy life expectancy and reduction of health disparities; (2) prevention of onset and progression of NCDs; (3) improvement of functions necessary for social life; (4) improvement in social environments to support and protect health; and (5) lifestyle improvements.

In Chapter 1, the targets for extending healthy life expectancy and reducing health inequality are defined as increasing healthy life expectancy beyond the increase in life expectancy and reducing inequality in healthy life expectancy between prefectures. The interim evaluation of the second term of HJ21 performed in 2018 judged that both targets have improved, which is the opposite of the GBD 2019 findings as described above. However, this difference can be attributed mainly to the method used to calculate healthy life expectancy.^20^ The interim evaluation used healthy life expectancy based on an individual's subjective judgment of health status, while the GBD uses objective healthy life expectancy based on YLDs.^20^

Other targets are not indexed by health loss taking into account both deaths and disability simultaneously, such as DALYs, but are mostly set in terms of epidemiological, clinical outcomes, such as deaths per population, number and proportion of patients, and population-level averages. Chapter 2 covers four chronic diseases as priority areas, including cancer, cardiovascular disease, diabetes, and chronic obstructive pulmonary disease (COPD) (Supplementary Table 8). During the interim evaluation of the second term of HJ21, seven of the 14 target items in this chapter were judged to have improved (although only three were considered likely to be achievable), especially for cancer and cardiovascular disease. However, improvements have not been acknowledged for most targets set for diabetes and COPD prevention.^21^^,^^22^ As mentioned above, according to GBD 2019, health losses attributable to diabetes have substantially increased since 2013; increases have also been noted for COPD (DALYs per population increased by 8.86%). In the interim evaluation, cardiovascular disease was evaluated based on age-adjusted mortality. According to the GBD 2019, DALYs and deaths per population for stroke and ischemic heart disease increased between 2013 and 2019 (Figure 1), but when age-adjusted (when the effect of aging was eliminated), no increase was observed (data not shown in tables or figures), which is consistent with the interim assessment.

Except for high BMI, important metabolic risk factors as highlighted in GBD 2019 are set as target items in this chapter. Blood pressure^23^^,^^24^ and LDL cholesterol control^25^ are considered for cardiovascular disease, and glycemic control^26^ is considered for diabetes. Improvement in all but LDL cholesterol was recognized in the interim evaluation. However, according to the GBD 2019, the disease burdens from these risk factors were estimated to increase between 2013 and 2019. This discrepancy may be due to the fact that the interim evaluation assessed the average value of clinical laboratory measurements or the percentage of threshold values exceeded, rather than health loss indicators such as the burden of disease as assessed by the GBD, and did not take into account differences in age groups to evaluate these risk factors.

Of the other important NCDs in terms of DALYs highlighted above and elsewhere,^27^ low back pain^28^^,^^29^ and Alzheimer's disease^30^, ^31^, ^32^ are each covered as a single target item within the priority areas of health for older adults in Chapter 3. Age-related hearing loss^33^^,^^34^ is not considered anywhere, which is in the top 10 contributors to current health losses from NCDs in DALYs in Japan, as shown above. Regarding Alzheimer's disease, unlike other target items, the target was not epidemiological, clinical outcomes, but the improvement in the identification of the population at high risk of cognitive decline.^21^ However, because of the difficulty in evaluating that target in the interim evaluation, it was proposed that the target be changed to increase the number of “dementia supporters”.^21^ A dementia supporter is a person who has a proper understanding of dementia and supports people with dementia. The certification is granted to those who have attended dementia supporter training courses held in municipalities and workplaces. For low back pain, the target item was to reduce the percentage of older people with leg and back pain, and improvement was observed, which is consistent with the GBD 2019 findings as described above.

Furthermore, for lifestyle improvements, six lifestyles with 22 targets are considered as priority areas in Chapter 5: nutrition and diet, physical activity and exercise, rest, smoking, alcohol use, and dental and oral health (Supplementary Table 9). Except for nutrition and diet and physical activity and exercise, improvement was acknowledged in more than half of the targets.^21^^,^^22^ The interim evaluation concluded that improvement was not observed in decreasing alcohol consumption.^21^ As part of the target, a statistical reduction in dietary salt has been observed. High BMI^35^ is covered as a target item of the nutrition and diet area and no improvement was observed. These findings are consistent with the GBD 2019 findings as described above.

## More public health efforts are needed to address critical risk factors for the rising burden of NCDs

As disability due to NCDs becomes a large part of health loss and a significant component of healthcare expenditures, finding new and more effective interventions has become an urgent and compelling challenge. As the population ages rapidly, the demand for healthcare services that can manage disabling outcomes and chronic conditions will require a stronger government commitment, accountability backed by better quality data, and a concerted effort to prioritize the most vulnerable populations.^36^ In the face of competing priorities for scarce resources, DALYs, a comprehensive health loss metric that assesses both deaths and disability and thus may reflect public health needs better than other health indicators, could be considered an important benchmark in determining research and action priorities for decision making.

While the need to align investments in health research with public health needs is one of the most important public health challenges in Japan, as it is worldwide, prior research provides a snapshot of the limited alignment between publicly competitive disease-specific funding and health losses in Japan. This calls for greater management over the allocation of scarce resources for health research and action.^37^

In Japan, smoking has a great influence on the population's health. While there is solid scientific evidence of its harmful effects, as of 2019 approximately 26% of men and 7% of women still smoke daily.^38^ Japan is a global laggard in tobacco control and does not meet standards set by the World Health Organization's (WHO) Framework Convention on Tobacco Control (FCTC),^39^ including high prices for tobacco products,^40^ plain packaging,^40^ and smoke-free public spaces.^41^ Such favourable circumstances for smokers may indicate that tobacco taxes were once one of the most important sources of government revenue and that the current ruling Liberal Democratic Party (LDP) has historically had a strong relationship with the tobacco industry.^41^^,^^42^ It is worth noting that tobacco smoking is still the largest contributor to health loss in terms of both DALYs and deaths among men. The WHO and the scientific community have recognized that excise taxes are an effective means of curbing the consumption of tobacco (and other significant health risks, such as alcohol) and expect that financial measures will provide an attractive complementary opportunity to improve population health by modifying risk factors without incurring additional costs.^43^^,^^44^

There is still room for improvement in high blood pressure control and prevention in Japan.^45^ Community and clinical efforts, such as early detection, lifestyle modification, and effective treatment of high blood pressure may reduce cardiovascular mortality and increase life expectancy, as has been the case in the last 20 years.^1^ Enhancing adherence to standard clinical guideline recommendations^46^ in general practice through ongoing medical education may be key to expanding the effective coverage of outpatient services and ensuring patient compliance.^47^

A comprehensive prevention package, including lifestyle and dietary modification and expanded, effective coverage of hypoglycemic agents, is needed to reduce the impact of several NCD risk factors, including high fasting plasma glucose, high BMI, high LDL cholesterol, alcohol use, high-sodium diets, and diets low in whole grains. Several previous studies have forecasted future changes in health loss in DALYs in Japan to which dietary risk factors contribute, including high salt intake, under various future intake scenarios.^48^, ^49^, ^50^ Significant gaps in the estimates of future health loss between scenarios were identified, indicating that future policies targeting risk factors in a population may have a significant impact on future trajectories of risk factor profiles in Japan and associated health losses.

Further, Japan has been cited as one of the countries with the lowest confidence in vaccines in the world.^51^ The MHLW suspended proactive recommendation of the HPV vaccination in 2013 due to unconfirmed reports of adverse events after vaccination, which were covered extensively by the media.^52^ In fact, there was a substantial decrease in the HPV vaccination rate in Japan from 68.4–74.0% in the 1994–98 birth cohort to 0.6% in the 2000 birth cohort. The second term of HJ21 includes cancer screening coverage (including for cervical cancer) as a target item for the cancer area in Chapter 2, but does not include HPV vaccination coverage. However, the situation regarding the HPV vaccine has changed significantly in 2021, and the MHLW decided that proactive recommendation of the HPV vaccination will be resumed in April 2022. It is expected that this will be reflected in the third term of HJ21.

Several NCDs and their underlying risk factors highlighted above are known to be associated with an increased risk of serious, negative health consequences due to COVID-19.^53^, ^54^, ^55^ While NCDs are spreading globally, the lack of sufficient public health efforts to control the increase in potentially preventable risk factors has left the Japanese people vulnerable to acute health crises, such as the current COVID-19 pandemic. Urgent measures need to be taken to combat the syndemic of NCDs and COVID-19, which will ensure a healthier population, making the country more resilient to the threat of future pandemics.

It is important to note that the data we discussed in this paper on NCDs and risk factors relies on the GBD 2019, so we were not able to address those that were not included in the GBD. For example, in Chapter 5, rest is one of the priority areas for lifestyle improvement, and the target items are sleep and working hours: insufficient sleep and excessive working hours are known to be associated with health risks such as hypertension, other cardiovascular diseases, and diabetes^56^^,^^57^ but are not covered in the current GBD.

## Conclusions

Our study highlights that there has been a particularly large increase in exposure to several preventable metabolic and behavioural risk factors in the past few years, and these risks may have contributed to the increased burden of NCDs in Japan. Progress has been also limited in NCDs and their associated risk factors, which are covered as target items in the second term of HJ21. It is therefore imperative to strengthen public health efforts to make the country more resilient to future health crises such as the COVID-19 pandemic. As global lessons from tobacco and alcohol control suggest, governments may need to take concerted action through regulation, taxation, and subsidies when there is a significant risk to population health, or when dramatic health improvements can be expected from risk improvement. In addition, along with rising longevity and population aging, disability due to NCDs accounts for the majority of health loss, and the demand for health services that can manage disabling outcomes and chronic diseases will require a stronger commitment from governments. DALYs, which assess disability as well as death, is a comprehensive health loss indicator and may well reflect public health needs; therefore, it should be considered an important metric for future research and decision-making.

## Declaration interests

The authors declare no conflict of interest.

## Appendix Supplementary materials

Image, application 1
