# Supplementary material for: Toward a third term of Health Japan 21 – implications from the rise in non-communicable disease burden and highly preventable risk factors
Source: Lancet Reg Health West Pac. 2022 Jan 23;21:100377. doi: 10.1016/j.lanwpc.2021.100377 (PMC8783949; doi:10.1016/j.lanwpc.2021.100377)
Supplement: Supplementary file 1 [file mmc1.pdf]

Supplementary Figure 1: Causes of all-ages DALYs and deaths per 100,000 population in Japan in 2013 and 2019 for women

	Leading causes in 2013	DALYs per 100,000 (95% uncertainty intervals)		Leading causes in 2019	DALYs per 100,000 (95% uncertainty intervals)
1	Low back pain	2175.42 (1547.27–2933.42)	1	Alzheimer's disease and other dementias	2114.20 (1017.42–4077.00)
2	Alzheimer's disease and other dementias	1783.12 (828.55–3520.77)	2	Low back pain	2036.82 (1441.98–2742.03)
3	Stroke	1743.54 (1443.39–1969.91)	3	Stroke	1876.28 (1548.04–2136.23)
4	Other musculoskeletal disorders	1069.38 (733.44–1476.78)	4	Ischemic heart disease	1117.57 (867.73–1263.47)
5	Ischemic heart disease	1048.92 (819.00–1174.99)	5	Other musculoskeletal disorders	1112.66 (770.30–1550.65)
6	Osteoarthritis	1009.45 (515.02–2023.51)	6	Osteoarthritis	963.58 (483.94–1922.29)
7	Age-related and other hearing loss	777.53 (532.02–1083.42)	7	Age-related and other hearing loss	855.86 (589.74–1185.30)
8	Colon and rectum cancer	643.84 (547.63–699.12)	8	Colon and rectum cancer	678.56 (565.23–746.21)
9	Headache disorders	625.43 (170.01–1293.63)	9	Falls	658.81 (493.45–875.19)
10	Breast cancer	620.56 (565.84–665.12)	10	Headache disorders	612.00 (169.21–1262.94)
11	Falls	566.82 (424.81–750.86)	11	Diabetes mellitus	611.54 (442.39–819.05)
12	Tracheal, bronchus, and lung cancer	549.95 (463.62–595.73)	12	Breast cancer	603.33 (543.13–659.55)
13	Chronic kidney disease	538.72 (450.71–611.67)	13	Tracheal, bronchus, and lung cancer	581.82 (481.66–638.95)
14	Depressive disorders	523.92 (369.59–711.78)	14	Chronic kidney disease	555.92 (457.87–637.00)
15	Diabetes mellitus	499.22 (367.20–663.65)	15	Depressive disorders	523.73 (369.72–708.23)
16	Endocrine, metabolic, blood, and immune disorders	485.04 (341.58–659.93)	16	Endocrine, metabolic, blood, and immune disorders	490.33 (342.49–671.13)
17	Gynecological diseases	484.81 (333.87–690.76)	17	Oral disorders	482.92 (295.91–748.36)
18	Self-harm	463.44 (444.15–478.90)	18	Gynecological diseases	476.46 (329.32–676.29)
19	Stomach cancer	462.95 (391.53–501.85)	19	Stomach cancer	446.97 (373.70–491.04)
20	Oral disorders	443.22 (269.07–696.53)	20	Pancreatic cancer	415.42 (346.15–456.46)
21	Pancreatic cancer		21	Self-harm	

	Leading causes in 2013	Deaths per 100,000 (95% uncertainty intervals)		Leading causes in 2019	Deaths per 100,000 (95% uncertainty intervals)
1	Alzheimer's disease and other dementias	143.15 (41.43–325.78)	1	Alzheimer's disease and other dementias	174.06 (51.39–395.76)
2	Stroke	103.98 (75.97–120.11)	2	Stroke	119.92 (86.70–140.14)
3	Ischemic heart disease	95.11 (69.15–109.74)	3	Ischemic heart disease	108.26 (78.72–126.45)
4	Colon and rectum cancer	40.98 (31.82–45.87)	4	Colon and rectum cancer	46.80 (35.80–53.05)
5	Tracheal, bronchus, and lung cancer	35.99 (28.12–40.18)	5	Tracheal, bronchus, and lung cancer	41.02 (31.41–46.28)
6	Chronic kidney disease	31.55 (22.86–36.41)	6	Chronic kidney disease	36.60 (25.93–43.01)
7	Stomach cancer	30.59 (23.74–34.29)	7	Stomach cancer	32.17 (24.79–36.41)
8	Pancreatic cancer	25.13 (19.97–27.87)	8	Pancreatic cancer	28.78 (22.53–32.45)
9	Breast cancer	23.53 (20.36–25.25)	9	Breast cancer	24.29 (20.41–26.39)
10	Liver cancer	17.53 (13.53–19.64)	10	Chronic obstructive pulmonary disease	20.19 (13.37–30.32)
11	Chronic obstructive pulmonary disease	17.28 (11.78–25.84)	11	Liver cancer	18.91 (14.67–21.47)
12	Cirrhosis and other chronic liver diseases	16.29 (12.70–18.97)	12	Cirrhosis and other chronic liver diseases	17.45 (13.23–20.84)
13	Gallbladder and biliary tract cancer	15.63 (9.99–18.31)	13	Non-rheumatic valvular heart disease	17.08 (10.78–20.54)
14	Non-rheumatic valvular heart disease	13.70 (8.86–16.25)	14	Gallbladder and biliary tract cancer	17.05 (10.61–20.21)
15	Self-harm	12.90 (11.81–13.49)	15	Aortic aneurysm	15.38 (11.40–17.78)
16	Aortic aneurysm	12.40 (9.16–14.15)	16	Hypertensive heart disease	14.99 (9.45–18.25)
17	Hypertensive heart disease	11.89 (7.65–14.28)	17	Self-harm	11.45 (10.34–12.21)
18	Parkinson's disease	10.07 (7.44–11.52)	18	Parkinson's disease	11.40 (8.49–13.11)
19	Atrial fibrillation and flutter	8.72 (6.01–12.82)	19	Atrial fibrillation and flutter	10.79 (7.37–16.42)
20	Non-Hodgkin lymphoma	8.38 (6.53–9.37)	20	Non-Hodgkin lymphoma	9.26 (7.12–10.46)

Increase of consistent in ranking order
Decrease in ranking order
Non-communicable diseases excluding injuries
Injuries

* Data were obtained from the GBD results tool: http://ghdx.healthdata.org/gbd-results-tool.

Supplementary Figure 2: Causes of all-ages DALYs and deaths per 100,000 population in Japan in 2013 and 2019 for men

	Leading causes in 2013	DALYs per 100,000 (95% uncertainty intervals)		Leading causes in 2019	DALYs per 100,000 (95% uncertainty intervals)
1	Stroke	1930.97 (1759.64–2072.76)	1	Stroke	1984.86 (1786.85–2148.56)
2	Ischemic heart disease	1801.08 (1673.66–1873.77)	2	Ischemic heart disease	1773.16 (1628.78–1866.40)
3	Tracheal, bronchus, and lung cancer	1604.37 (1492.91–1658.64)	3	Tracheal, bronchus, and lung cancer	1551.57 (1430.69–1634.62)
4	Low back pain	1365.72 (955.45–1859.41)	4	Low back pain	1243.54 (872.57–1682.90)
5	Self-harm	1198.76 (1180.77–1215.39)	5	Alzheimer's disease and other dementias	1085.20 (468.30–2428.90)
6	Stomach cancer	1035.80 (966.65–1071.40)	6	Self-harm	1010.12 (975.99–1045.07)
7	Colon and rectum cancer	954.81 (895.19–993.72)	7	Colon and rectum cancer	980.82 (905.03–1036.85)
8	Alzheimer's disease and other dementias	886.66 (367.94–2020.98)	8	Stomach cancer	963.85 (883.91–1015.88)
9	Age-related and other hearing loss	805.29 (546.95–1154.57)	9	Age-related and other hearing loss	881.57 (600.75–1252.22)
10	Chronic obstructive pulmonary disease	790.40 (668.55–926.99)	10	Chronic obstructive pulmonary disease	855.13 (716.30–1015.05)
11	Other musculoskeletal disorders	758.71 (519.08–1061.39)	11	Diabetes mellitus	829.51 (607.74–1089.87)
12	Diabetes mellitus	728.61 (539.93–952.68)	12	Other musculoskeletal disorders	745.68 (510.11–1042.24)
13	Falls	659.88 (519.69–843.04)	13	Falls	715.22 (561.00–918.62)
14	Chronic kidney disease	646.96 (580.31–711.06)	14	Chronic kidney disease	649.69 (578.68–717.97)
15	Liver cancer	641.67 (603.54–662.50)	15	Liver cancer	624.05 (577.29–665.94)
16	Cirrhosis and other chronic liver diseases	552.44 (526.46–581.05)	16	Pancreatic cancer	535.09 (494.17–562.30)
17	Pancreatic cancer	528.46 (498.94–544.99)	17	Cirrhosis and other chronic liver diseases	520.86 (492.14–554.76)
18	Oral disorders	402.31 (238.02–630.28)	18	Oral disorders	442.77 (263.87–687.85)
19	Osteoarthritis	388.80 (197.22–784.72)	19	Osteoarthritis	421.32 (213.16–849.18)
20	Headache disorders	376.01 (131.77–740.00)	20	Prostate cancer	393.57 (307.46–516.01)
23	Prostate cancer		21	Headache disorders	

	Leading causes in 2013	Deaths per 100,000 (95% uncertainty intervals)		Leading causes in 2019	Deaths per 100,000 (95% uncertainty intervals)
1	Ischemic heart disease	104.31 (93.00–110.11)	1	Ischemic heart disease	111.87 (97.86–119.98)
2	Stroke	96.18 (85.10–102.14)	2	Stroke	106.14 (91.27–114.32)
3	Tracheal, bronchus, and lung cancer	91.73 (83.29–96.03)	3	Tracheal, bronchus, and lung cancer	94.95 (85.03–100.98)
4	Alzheimer's disease and other dementias	63.12 (16.06–167.91)	4	Alzheimer's disease and other dementias	81.63 (21.14–212.50)
5	Stomach cancer	57.62 (52.12–60.36)	5	Stomach cancer	57.95 (51.68–61.76)
6	Colon and rectum cancer	48.22 (43.84–50.35)	6	Colon and rectum cancer	53.12 (47.49–56.35)
7	Liver cancer	34.33 (31.49–35.77)	7	Chronic obstructive pulmonary disease	39.64 (30.27–50.09)
8	Chronic obstructive pulmonary disease	33.75 (26.35–42.03)	8	Liver cancer	35.53 (32.08–38.05)
9	Self-harm	31.74 (30.81–32.31)	9	Chronic kidney disease	34.28 (29.22–37.28)
10	Chronic kidney disease	30.66 (26.71–32.61)	10	Pancreatic cancer	29.88 (26.83–31.73)
11	Pancreatic cancer	27.54 (25.27–28.66)	11	Self-harm	27.73 (26.41–28.84)
12	Cirrhosis and other chronic liver diseases	23.73 (21.97–25.73)	12	Prostate cancer	26.35 (19.65–33.03)
13	Prostate cancer	21.88 (16.19–27.37)	13	Cirrhosis and other chronic liver diseases	23.79 (21.69–26.24)
14	Esophageal cancer	17.79 (16.47–18.45)	14	Esophageal cancer	18.56 (16.88–19.86)
15	Gallbladder and biliary tract cancer	15.01 (10.04–16.31)	15	Gallbladder and biliary tract cancer	16.45 (10.85–18.28)
16	Aortic aneurysm	13.97 (12.45–14.79)	16	Aortic aneurysm	16.20 (14.14–17.65)
17	Interstitial lung disease and pulmonary sarcoidosis	12.34 (4.71–17.55)	17	Parkinson's disease	14.91 (12.83–16.20)
18	Parkinson's disease	12.33 (10.76–13.11)	18	Interstitial lung disease and pulmonary sarcoidosis	14.37 (5.23–20.94)
19	Falls	11.37 (9.84–12.10)	19	Falls	12.95 (11.03–14.09)
20	Non-Hodgkin lymphoma	11.05 (9.90–11.56)	20	Non-Hodgkin lymphoma	12.28 (10.91–13.33)

Increase of consistent in ranking order
Decrease in ranking order
Non-communicable diseases excluding injuries
Injuries

* Data were obtained from the GBD results tool: http://ghdx.healthdata.org/gbd-results-tool.

Supplementary Figure 3: All-ages DALYs rate (per 100,000 population) attributable to preventable behavioral and metabolic risk factors in 2013 and 2019 for both sexes combined

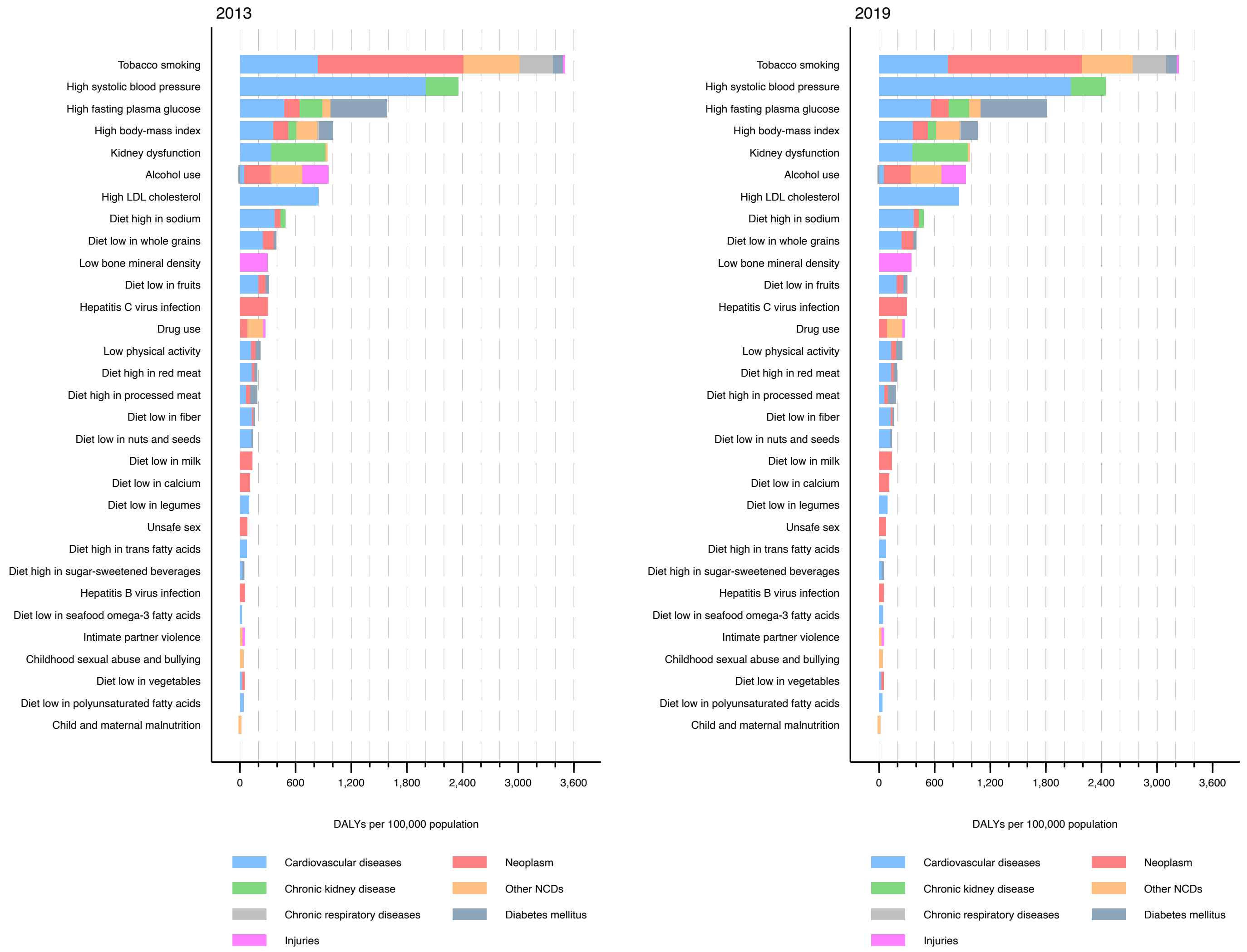

* Data, except for hepatitis B and C virus infections, were obtained from the GBD results tool: http://ghdx.healthdata.org/gbd-results-tool. DALYs: disability-adjusted life years; NCD: non-communicable disease; LDL: low-density lipoprotein. The order of risk factors is sorted by 2019 values.

Supplementary Figure 4: All-ages mortality rate (per 100,000) attributable to preventable behavioral and metabolic risk factors in 2013 and 2019 for both sexes combined

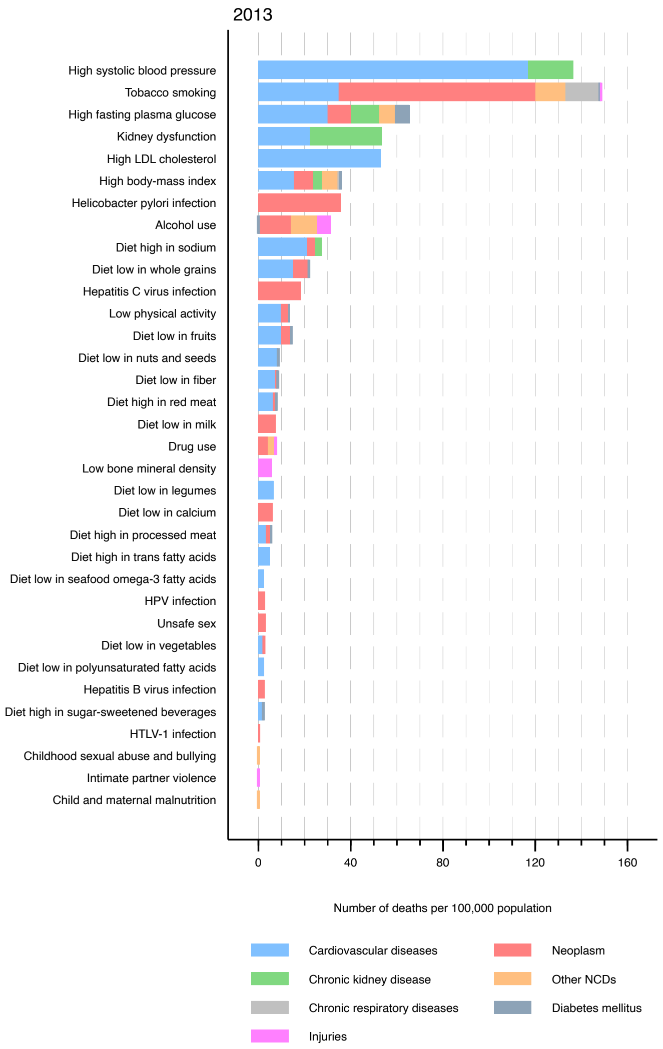

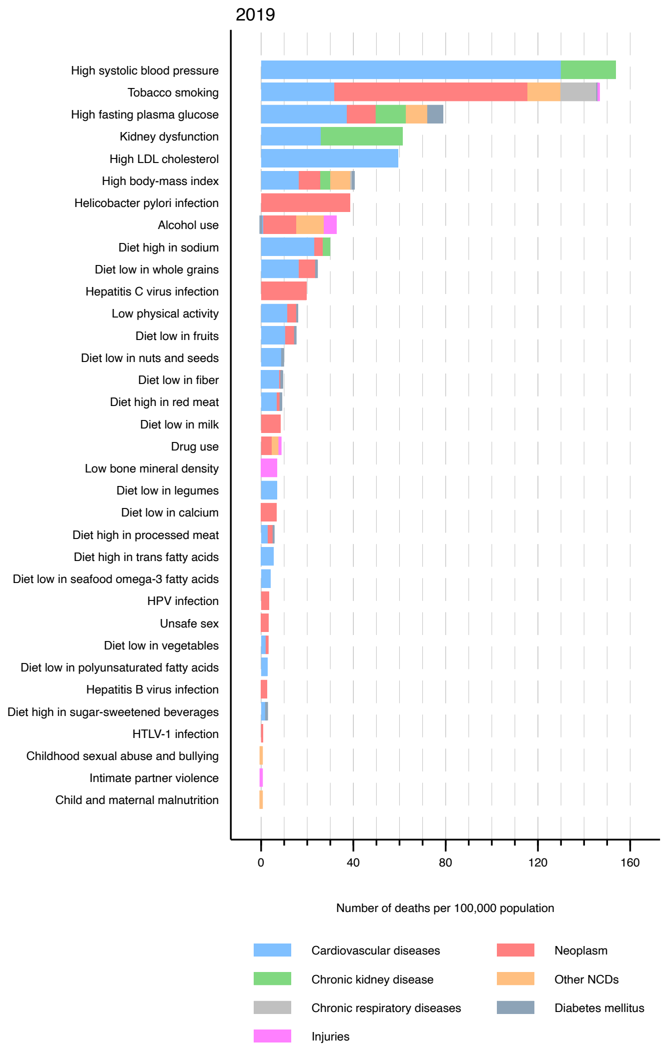

* Data, except for hepatitis B and C virus infections, *Helicobacter pylori* infection, HPV infection, and HTLV-1 infection, were obtained from the GBD results tool: http://ghdx.healthdata.org/gbd-results-tool. NCD: non-communicable disease; LDL: low-density lipoprotein; HPV: human papillomavirus; HTLV-1: human T-cell leukemia virus type 1. The order of risk factors is sorted by 2019 values.

Supplementary Figure 5: All-ages DALYs attributable to preventable behavioral and metabolic risk factors in 2013 and 2019 for women

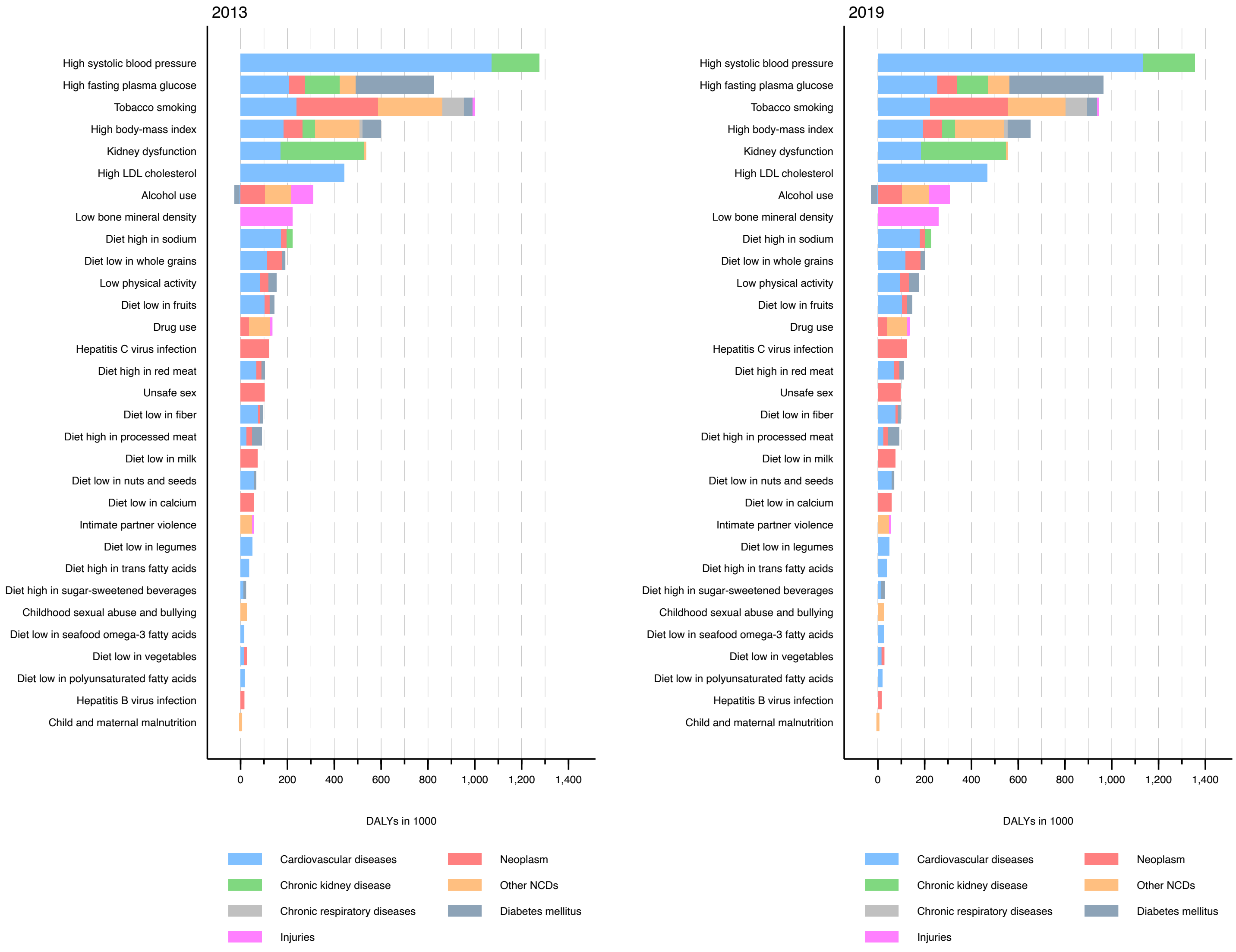

* Data, except for hepatitis B and C virus infections, were obtained from the GBD results tool: http://ghdx.healthdata.org/gbd-results-tool. DALYs: disability-adjusted life years; NCD: non-communicable disease; LDL: low-density lipoprotein. The order of risk factors is sorted by 2019 values.

Supplementary Figure 6: All-ages number of deaths attributable to preventable behavioral and metabolic risk factors in 2013 and 2019 for women

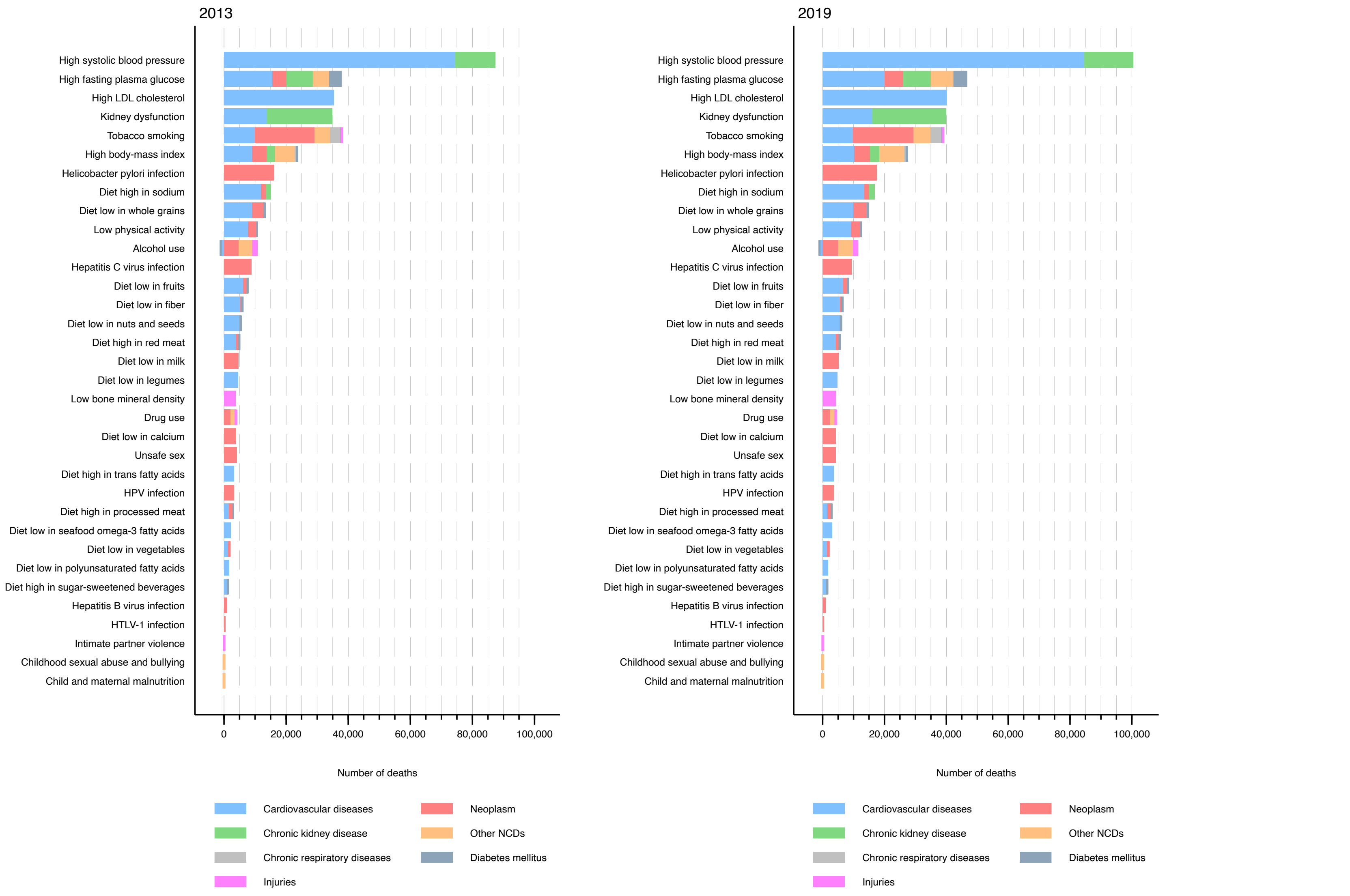

* Data, except for hepatitis B and C virus infections, *Helicobacter pylori* infection, HPV infection, and HTLV-1 infection, were obtained from the GBD results tool: http://ghdx.healthdata.org/gbd-results-tool. NCD: non-communicable disease; LDL: low-density lipoprotein; HPV: human papillomavirus; HTLV-1: human T-cell leukemia virus type 1. The order of risk factors is sorted by 2019 values.

Supplementary Figure 7: All-ages DALYs rate (per 100,000 population) attributable to preventable behavioral and metabolic risk factors in 2013 and 2019 for women

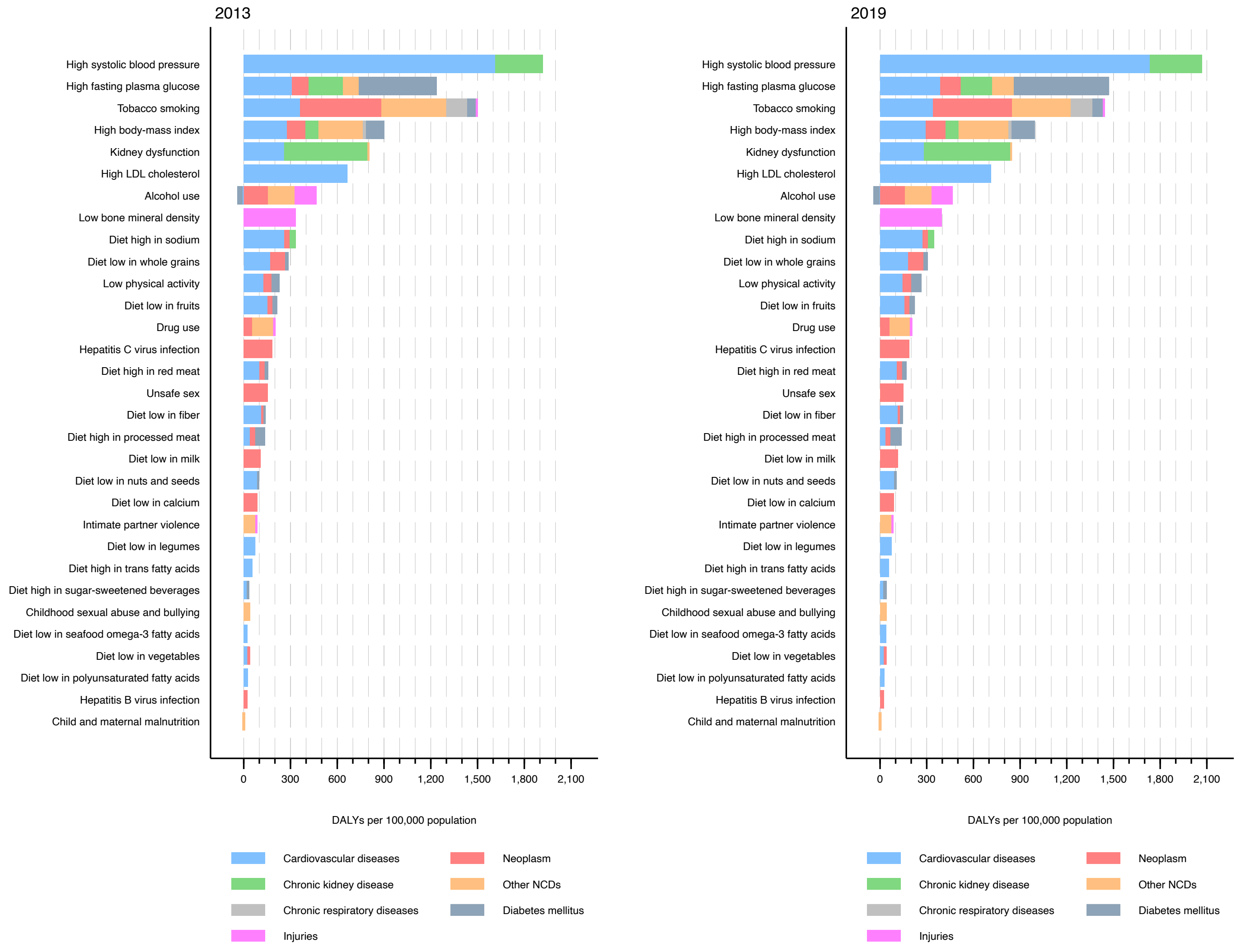

* Data, except for hepatitis B and C virus infections, were obtained from the GBD results tool: http://ghdx.healthdata.org/gbd-results-tool. DALYs: disability-adjusted life years; NCD: non-communicable disease; LDL: low-density lipoprotein. The order of risk factors is sorted by 2019 values.

Supplementary Figure 8: All-ages mortality rate (per 100,000) attributable to preventable behavioral and metabolic risk factors in 2013 and 2019 for women

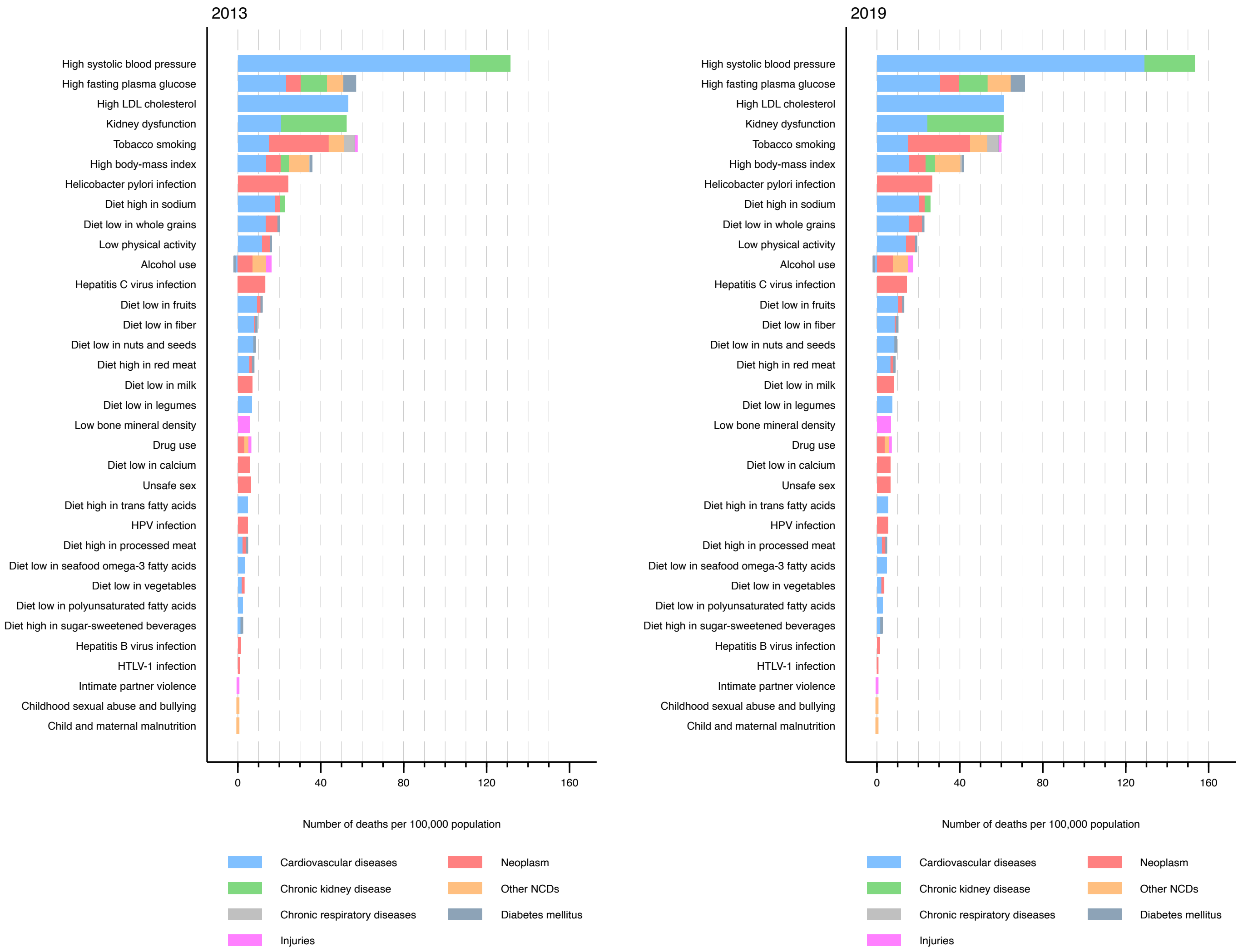

* Data, except for hepatitis B and C virus infections, *Helicobacter pylori* infection, HPV infection, and HTLV-1 infection, were obtained from the GBD results tool: http://ghdx.healthdata.org/gbd-results-tool. NCD: non-communicable disease; LDL: low-density lipoprotein; HPV: human papillomavirus; HTLV-1: human T-cell leukemia virus type 1. The order of risk factors is sorted by 2019 values.

Supplementary Figure 9: All-ages DALYs attributable to preventable behavioral and metabolic risk factors in 2013 and 2019 for men

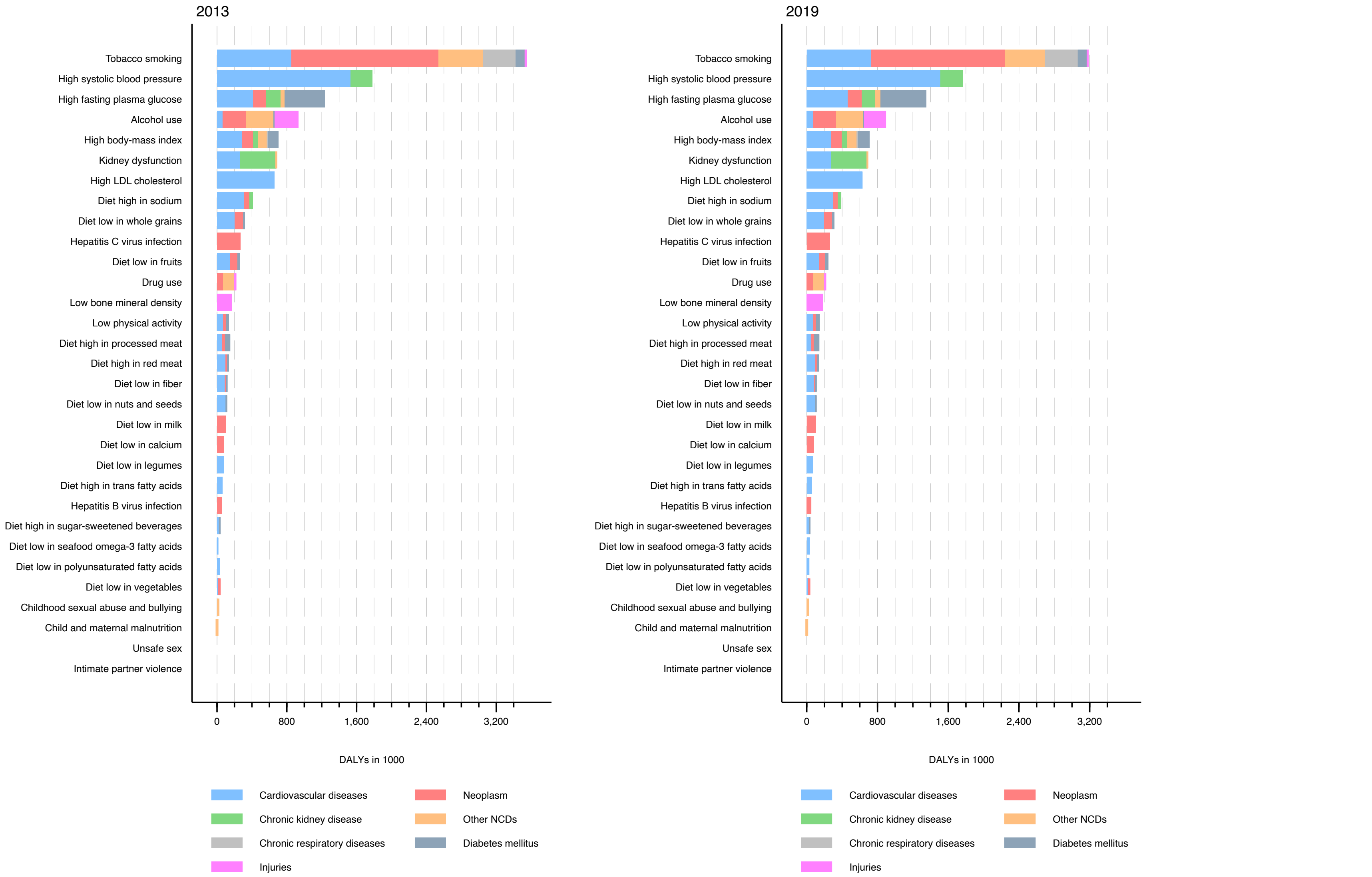

* Data, except for hepatitis B and C virus infections, were obtained from the GBD results tool: http://ghdx.healthdata.org/gbd-results-tool. DALYs: disability-adjusted life years; NCD: non-communicable disease; LDL: low-density lipoprotein. The order of risk factors is sorted by 2019 values.

Supplementary Figure 10: All-ages number of deaths attributable to preventable behavioral and metabolic risk factors in 2013 and 2019 for men

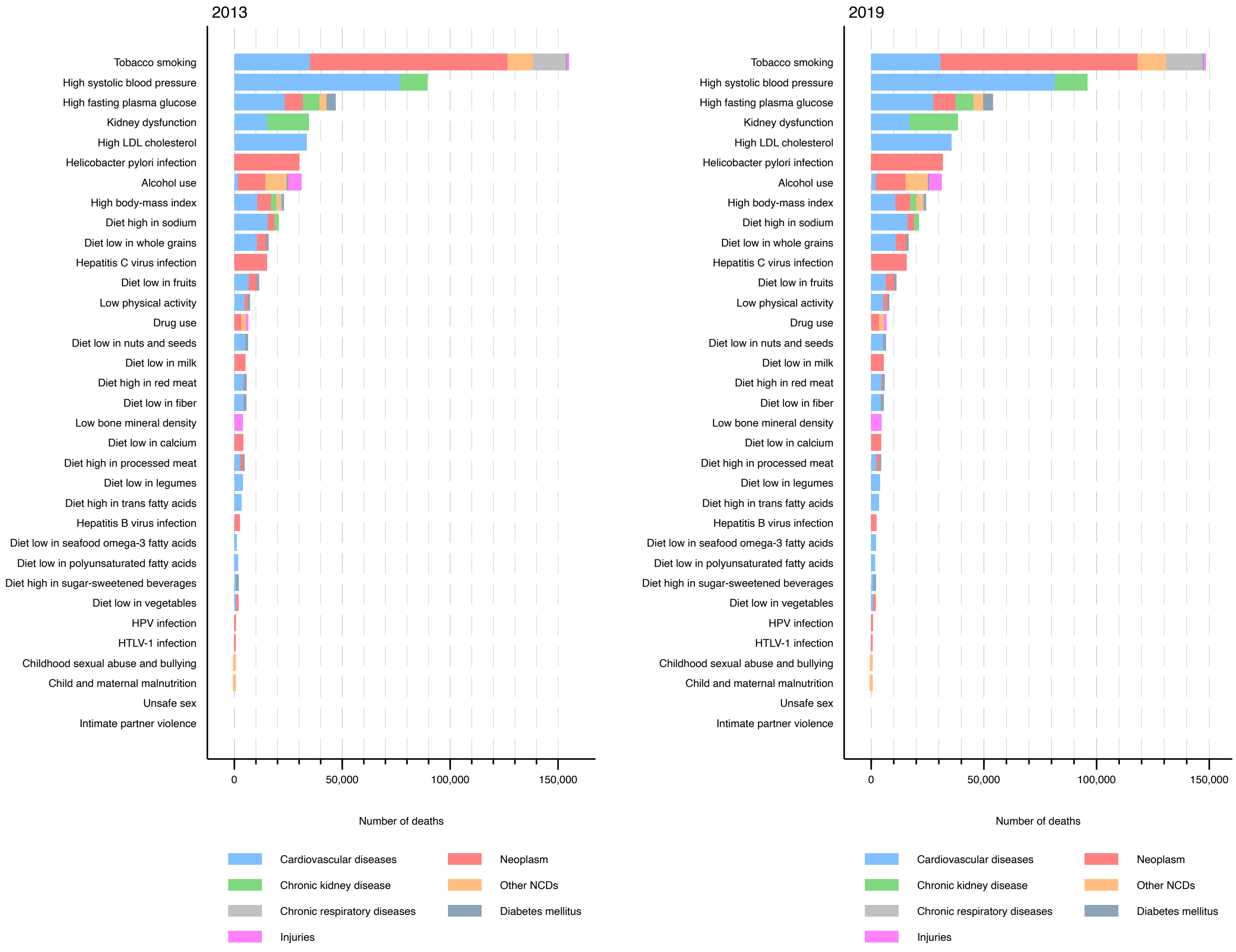

* Data, except for hepatitis B and C virus infections, *Helicobacter pylori* infection, HPV infection, and HTLV-1 infection, were obtained from the GBD results tool: http://ghdx.healthdata.org/gbd-results-tool. NCD: non-communicable disease; LDL: low-density lipoprotein; HPV: human papillomavirus; HTLV-1: human T-cell leukemia virus type 1. The order of risk factors is sorted by 2019 values.

Supplementary Figure 11: All-ages DALYs rate (per 100,000 population) attributable to preventable behavioral and metabolic risk factors in 2013 and 2019 for men

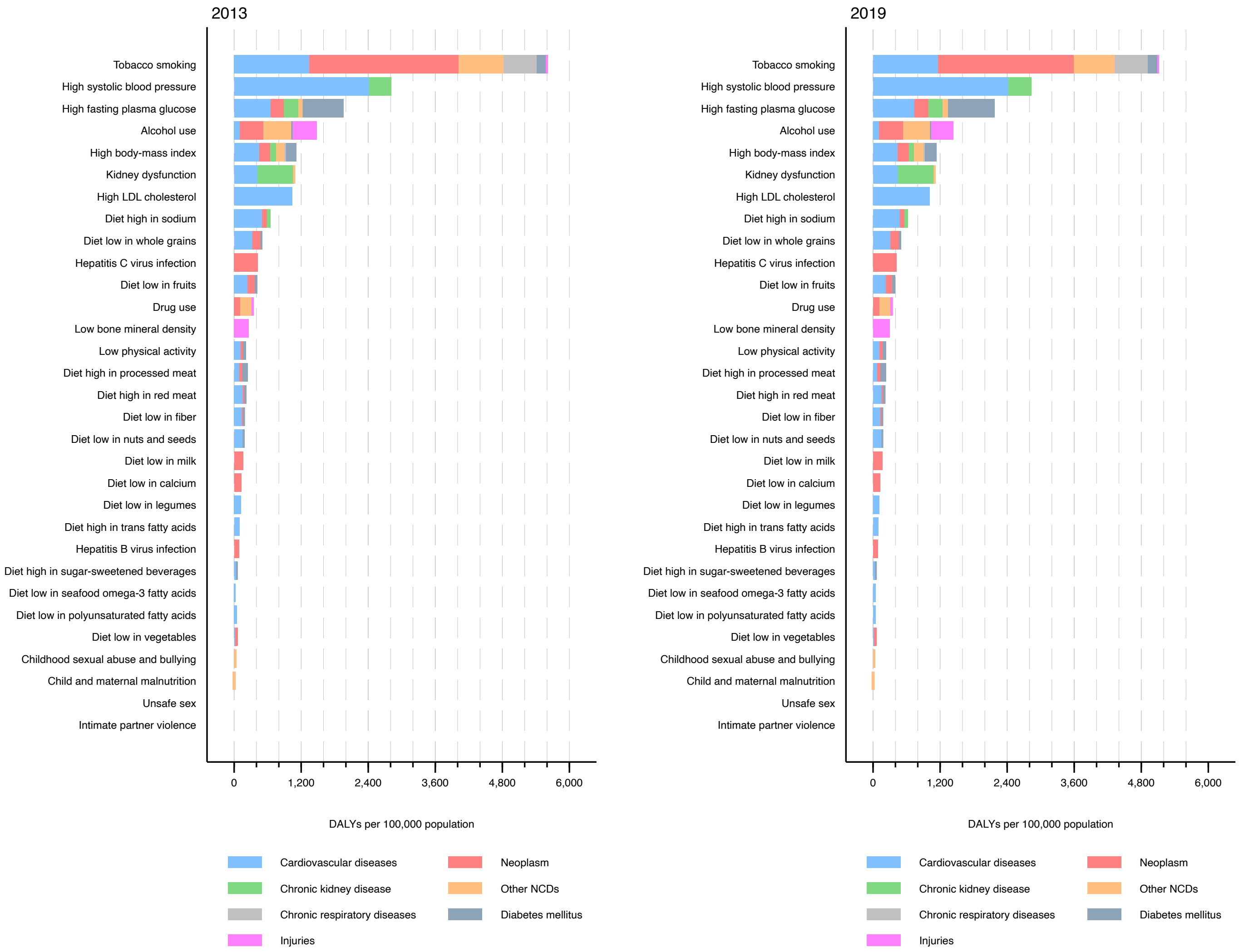

* Data, except for hepatitis B and C virus infections, were obtained from the GBD results tool: http://ghdx.healthdata.org/gbd-results-tool. DALYs: disability-adjusted life years; NCD: non-communicable disease; LDL: low-density lipoprotein. The order of risk factors is sorted by 2019 values.

Supplementary Figure 12: All-ages mortality rate (per 100,000) attributable to preventable behavioral and metabolic risk factors in 2013 and 2019 for men

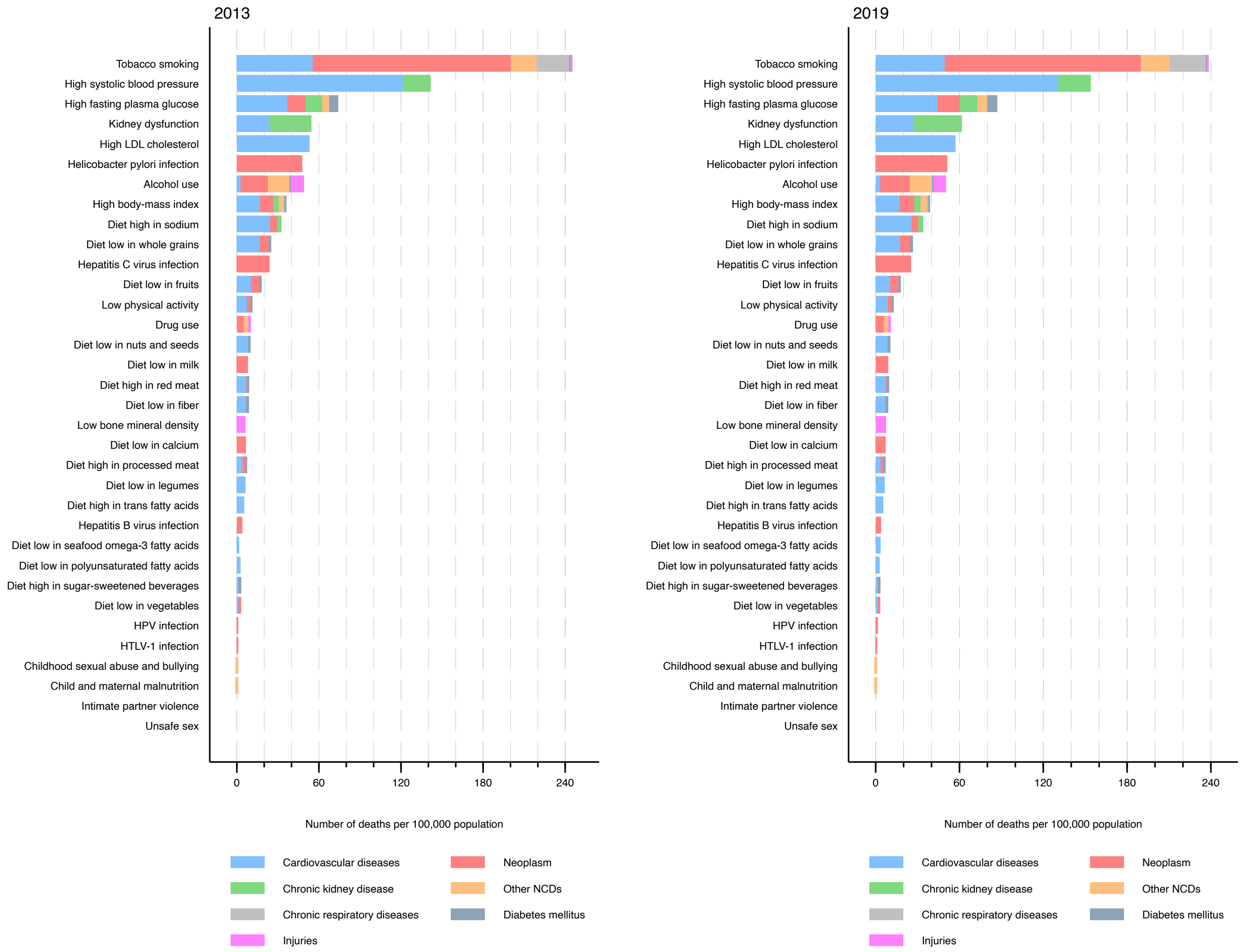

* Data, except for hepatitis B and C virus infections, *Helicobacter pylori* infection, HPV infection, and HTLV-1 infection, were obtained from the GBD results tool: http://ghdx.healthdata.org/gbd-results-tool. NCD: non-communicable disease; LDL: low-density lipoprotein; HPV: human papillomavirus; HTLV-1: human T-cell leukemia virus type 1. The order of risk factors is sorted by 2019 values.

Supplementary Table 1: Number of deaths from the Vital Statistics by ICD-10 codes in 2013 and 2019, and PAF of HPV infections and HYLV-1 infection in relation to each cause of death

			2013		2019		PAF*	
Risk factor	ICD-10	Cause	Women	Men	Women	Men	Women	Men
HPV infection	C01	Malignant neoplasm of base of tongue	5	23	6	37	46.0	46.0
HPV infection	C02	Malignant neoplasm of other and unspecified parts of tongue	501	729	533	821	4.3	4.3
HPV infection	C03	Malignant neoplasm of gum	612	623	653	482	4.3	4.3
HPV infection	C04	Malignant neoplasm of floor of mouth	70	198	64	179	4.3	4.3
HPV infection	C05	Malignant neoplasm of palate	44	43	54	51	4.3	4.3
HPV infection	C06	Malignant neoplasm of other and unspecified parts of mouth	231	206	273	242	4.3	4.3
HPV infection	C09	Malignant neoplasm of tonsil	1	24	4	19	46.0	46.0
HPV infection	C10	Malignant neoplasm of oropharynx	136	746	164	932	46.0	46.0
HPV infection	C21	Malignant neoplasm of anus and anal canal	180	170	230	235	88.0	88.0
HPV infection	C51	Malignant neoplasm of vulva	214	–	273	–	48.0	–
HPV infection	C52	Malignant neoplasm of vagina	127	–	143	–	78.0	–
HPV infection	C53	Malignant neoplasm of cervix uteri	2682	–	2955	–	100.0	–
HPV infection	C60	Malignant neoplasm of penis	–	139	–	159	–	51.0
HTLV-1 infection	C91.5	Adult T-cell lymphoma/leukaemia [HTLV-1-associated]	500	528	430	489	100.0	100.0

ICD-10: the 10th revision of the International Statistical Classification of Diseases and Related Health Problems; HPV: human papillomavirus; HTLV-1: human T-cell leukemia virus type 1; PAF: population attributable fraction.

* Data were extracted from the following previous studies: Plummer M, de Martel C, Vignat J, Ferlay J, Bray F, Franceschi S. Global burden of cancers attributable to infections in 2012: a synthetic analysis. *Lancet Glob Health* 2016; **4**(9): e609-16; Lin Y, Wang C, Kikuchi S, et al. Burden of cancer attributable to infection in Japan in 2015. GHM Open 2021; 1(2): 63-9.

Supplementary Table 2: All-age DALYs and number of deaths attributable to preventable behavioral and metabolic risk factors in 2013 and 2019 for both sexes combined

Year	Risk factors	Cause	DALYs	Number of deaths
2013	Alcohol use	Cardiovascular diseases	58930.80	747.95
2013	Alcohol use	Neoplasms	372916.88	17377.96
2013	Alcohol use	Other NCDs	439689.04	14985.15
2013	Alcohol use	Diabetes mellitus	-18698.70	-221.49
2013	Alcohol use	Injurie	364541.28	7756.45
2019	Alcohol use	Cardiovascular diseases	67447.16	1185.92
2019	Alcohol use	Neoplasms	368657.94	18285.47
2019	Alcohol use	Other NCDs	425555.41	15244.11
2019	Alcohol use	Diabetes mellitus	-21652.40	-234.78
2019	Alcohol use	Injurie	334562.81	7073.53
2013	Child and maternal malnutrition	Other NCDs	389.55	4.39
2019	Child and maternal malnutrition	Other NCDs	303.68	3.42
2013	Childhood sexual abuse and bullying	Other NCDs	52949.51	74.86
2019	Childhood sexual abuse and bullying	Other NCDs	51834.78	73.60
2013	Diet high in processed meat	Cardiovascular diseases	86290.63	4207.19
2013	Diet high in processed meat	Neoplasms	55905.91	2671.80
2013	Diet high in processed meat	Diabetes mellitus	99888.66	834.81
2019	Diet high in processed meat	Cardiovascular diseases	73401.70	3845.18
2019	Diet high in processed meat	Neoplasms	52096.18	2639.37
2019	Diet high in processed meat	Diabetes mellitus	108947.52	815.24
2013	Diet high in red meat	Cardiovascular diseases	166580.08	8024.92
2013	Diet high in red meat	Neoplasms	37726.12	1832.93
2013	Diet high in red meat	Diabetes mellitus	35665.70	342.25
2019	Diet high in red meat	Cardiovascular diseases	168019.55	8745.72
2019	Diet high in red meat	Neoplasms	38566.11	2015.55
2019	Diet high in red meat	Diabetes mellitus	42862.12	374.83
2013	Diet high in sodium	Cardiovascular diseases	486884.06	27348.39
2013	Diet high in sodium	Neoplasms	79943.82	4684.18
2013	Diet high in sodium	Chronic kidney disease	65992.77	3511.16
2019	Diet high in sodium	Cardiovascular diseases	479427.88	29506.63
2019	Diet high in sodium	Neoplasms	73464.70	4677.41
2019	Diet high in sodium	Chronic kidney disease	64716.29	3903.45
2013	Diet high in sugar-sweetened beverages	Cardiovascular diseases	37259.29	2571.72
2013	Diet high in sugar-sweetened beverages	Diabetes mellitus	24626.48	256.03
2019	Diet high in sugar-sweetened beverages	Cardiovascular diseases	38328.11	2857.54
2019	Diet high in sugar-sweetened beverages	Diabetes mellitus	30959.07	290.44
2013	Diet high in trans fatty acids	Cardiovascular diseases	97283.50	6480.17
2019	Diet high in trans fatty acids	Cardiovascular diseases	95911.49	6984.21
2013	Diet low in calcium	Neoplasms	139603.84	8027.69
2019	Diet low in calcium	Neoplasms	139003.97	8636.08
2013	Diet low in fiber	Cardiovascular diseases	169112.22	9567.80
2013	Diet low in fiber	Neoplasms	20735.14	1208.50
2013	Diet low in fiber	Diabetes mellitus	22174.85	246.30

2019	Diet low in fiber	Cardiovascular diseases	161553.47	9989.71
2019	Diet low in fiber	Neoplasms	20500.04	1296.40
2019	Diet low in fiber	Diabetes mellitus	25319.61	258.91
2013	Diet low in fruits	Cardiovascular diseases	257288.81	12820.94
2013	Diet low in fruits	Neoplasms	99799.90	5471.86
2013	Diet low in fruits	Diabetes mellitus	49687.80	499.72
2019	Diet low in fruits	Cardiovascular diseases	245154.25	13305.56
2019	Diet low in fruits	Neoplasms	91451.66	5391.82
2019	Diet low in fruits	Diabetes mellitus	54886.55	508.50
2013	Diet low in legumes	Cardiovascular diseases	127031.62	8447.49
2019	Diet low in legumes	Cardiovascular diseases	118017.91	8763.29
2013	Diet low in milk	Neoplasms	174654.22	9787.92
2019	Diet low in milk	Neoplasms	178533.45	10793.89
2013	Diet low in nuts and seeds	Cardiovascular diseases	160334.44	10996.69
2013	Diet low in nuts and seeds	Diabetes mellitus	19111.75	219.17
2019	Diet low in nuts and seeds	Cardiovascular diseases	157237.62	11794.82
2019	Diet low in nuts and seeds	Diabetes mellitus	22340.00	235.86
2013	Diet low in polyunsaturated fatty acids	Cardiovascular diseases	49921.66	3246.76
2019	Diet low in polyunsaturated fatty acids	Cardiovascular diseases	48406.09	3442.81
2013	Diet low in seafood omega-3 fatty acids	Cardiovascular diseases	28727.66	3181.22
2019	Diet low in seafood omega-3 fatty acids	Cardiovascular diseases	55854.02	5257.55
2013	Diet low in vegetables	Cardiovascular diseases	46426.44	2967.01
2013	Diet low in vegetables	Neoplasms	4235.23	230.03
2019	Diet low in vegetables	Cardiovascular diseases	45484.98	3207.28
2019	Diet low in vegetables	Neoplasms	4107.70	240.51
2013	Diet low in whole grains	Cardiovascular diseases	318843.53	19572.02
2013	Diet low in whole grains	Neoplasms	155385.27	8625.96
2013	Diet low in whole grains	Diabetes mellitus	34764.11	364.23
2019	Diet low in whole grains	Cardiovascular diseases	313456.31	20999.52
2019	Diet low in whole grains	Neoplasms	158507.73	9487.80
2019	Diet low in whole grains	Diabetes mellitus	40497.85	389.97
2013	Drug use	Neoplasms	106501.30	5411.86
2013	Drug use	Other NCDs	228993.69	4216.93
2013	Drug use	Injurie	9258.50	189.70
2019	Drug use	Neoplasms	111888.73	6057.20
2019	Drug use	Other NCDs	222359.04	4310.66
2019	Drug use	Injurie	7762.70	161.79
2013	HPV infection	Neoplasms	NA	3832.82
2019	HPV infection	Neoplasms	NA	4366.53
2013	HTLV-1 infection	Neoplasms	NA	1028.00
2019	HTLV-1 infection	Neoplasms	NA	919.00
2013	*Helicobacter pylori* infection	Neoplasms	NA	46272.78
2019	*Helicobacter pylori* infection	Neoplasms	NA	49283.78
2013	Hepatitis B virus infection	Neoplasms	71950.26	3407.12
2019	Hepatitis B virus infection	Neoplasms	67538.50	3368.15
2013	Hepatitis C virus infection	Neoplasms	391420.16	23930.62

2019	Hepatitis C virus infection	Neoplasms	383625.19	25051.50
2013	High LDL cholesterol	Cardiovascular diseases	1098180.88	68788.78
2019	High LDL cholesterol	Cardiovascular diseases	1098334.88	75782.03
2013	High body-mass index	Cardiovascular diseases	471109.91	19826.05
2013	High body-mass index	Neoplasms	204838.77	10894.04
2013	High body-mass index	Chronic kidney disease	116647.45	5043.33
2013	High body-mass index	Other NCDs	293028.75	9392.42
2013	High body-mass index	Chronic respiratory diseases	22690.56	232.16
2013	High body-mass index	Diabetes mellitus	193850.38	1445.98
2019	High body-mass index	Cardiovascular diseases	466874.38	21024.39
2019	High body-mass index	Neoplasms	207154.09	11740.97
2019	High body-mass index	Chronic kidney disease	114633.69	5615.01
2019	High body-mass index	Other NCDs	321541.23	11681.06
2019	High body-mass index	Chronic respiratory diseases	23452.75	250.30
2019	High body-mass index	Diabetes mellitus	228163.19	1510.36
2013	High fasting plasma glucose	Cardiovascular diseases	621306.38	38848.36
2013	High fasting plasma glucose	Neoplasms	216978.36	13171.44
2013	High fasting plasma glucose	Chronic kidney disease	313019.53	16110.77
2013	High fasting plasma glucose	Other NCDs	115394.76	8497.74
2013	High fasting plasma glucose	Diabetes mellitus	792262.38	8190.04
2019	High fasting plasma glucose	Cardiovascular diseases	717172.38	47646.45
2019	High fasting plasma glucose	Neoplasms	243409.91	15793.96
2019	High fasting plasma glucose	Chronic kidney disease	286796.81	16892.76
2019	High fasting plasma glucose	Other NCDs	153133.70	11691.59
2019	High fasting plasma glucose	Diabetes mellitus	917207.31	8784.08
2013	High systolic blood pressure	Cardiovascular diseases	2601975.75	151478.14
2013	High systolic blood pressure	Chronic kidney disease	452756.88	25484.87
2019	High systolic blood pressure	Cardiovascular diseases	2645609.50	166023.95
2019	High systolic blood pressure	Chronic kidney disease	477260.88	30360.66
2013	Intimate partner violence	Other NCDs	49350.63	0.00
2013	Intimate partner violence	Injurie	7787.61	71.09
2019	Intimate partner violence	Other NCDs	47613.55	0.00
2019	Intimate partner violence	Injurie	7821.76	66.85
2013	Kidney dysfunction	Cardiovascular diseases	437405.84	29030.57
2013	Kidney dysfunction	Chronic kidney disease	766899.56	40340.07
2013	Kidney dysfunction	Other NCDs	13050.30	0.00
2019	Kidney dysfunction	Cardiovascular diseases	461401.41	33093.30
2019	Kidney dysfunction	Chronic kidney disease	768793.31	45324.43
2019	Kidney dysfunction	Other NCDs	15305.70	0.00
2013	Low bone mineral density	Injurie	387035.31	7695.78
2019	Low bone mineral density	Injurie	445611.97	8920.78
2013	Low physical activity	Cardiovascular diseases	156742.94	12649.80
2013	Low physical activity	Neoplasms	64210.21	4243.01
2013	Low physical activity	Diabetes mellitus	67639.34	917.31
2019	Low physical activity	Cardiovascular diseases	169430.09	14615.16
2019	Low physical activity	Neoplasms	69114.70	4896.53

2019	Low physical activity	Diabetes mellitus	81577.53	1024.62
2013	Tobacco smoking	Cardiovascular diseases	1090582.62	45051.94
2013	Tobacco smoking	Neoplasms	2036222.25	110735.50
2013	Tobacco smoking	Other NCDs	783271.72	16873.07
2013	Tobacco smoking	Chronic respiratory diseases	466942.22	18555.35
2013	Tobacco smoking	Diabetes mellitus	138337.58	1114.51
2013	Tobacco smoking	Injurie	29190.91	542.00
2019	Tobacco smoking	Cardiovascular diseases	950184.62	40650.62
2019	Tobacco smoking	Neoplasms	1846583.88	107019.90
2019	Tobacco smoking	Other NCDs	699115.83	18153.41
2019	Tobacco smoking	Chronic respiratory diseases	463008.19	19833.55
2019	Tobacco smoking	Diabetes mellitus	146938.16	1069.25
2019	Tobacco smoking	Injurie	28246.11	511.17
2013	Unsafe sex	Neoplasms	103031.44	4172.02
2019	Unsafe sex	Neoplasms	97453.56	4210.49

* Data, except for hepatitis B and C virus infections, *Helicobacter pylori* infection, HPV infection, and HTLV-1 infection, were obtained from the GBD results tool: http://ghdx.healthdata.org/gbd-results-tool. DALYs: disability-adjusted life years; NCDs: non-communicable diseases; LDL: low-density lipoprotein; HPV: human papillomavirus; HTLV-1: human T-cell leukemia virus type 1. The order of the risk factors is sorted alphabetically.

Supplementary Table 3: All-age DALYs rate (per 100,000) and mortality rate (per 100,000) attributable to preventable behavioral and metabolic risk factors in 2013 and 2019 for both sexes combined

Year	Risk factors	Cause	DALYs rate	Mortality rate
2013	Alcohol use	Cardiovascular diseases	45.45	0.58
2013	Alcohol use	Neoplasms	287.62	13.40
2013	Alcohol use	Other NCDs	339.12	11.56
2013	Alcohol use	Diabetes mellitus	-14.42	-0.17
2013	Alcohol use	Injurie	281.16	5.98
2019	Alcohol use	Cardiovascular diseases	52.78	0.93
2019	Alcohol use	Neoplasms	288.49	14.31
2019	Alcohol use	Other NCDs	333.02	11.93
2019	Alcohol use	Diabetes mellitus	-16.94	-0.18
2019	Alcohol use	Injurie	261.81	5.54
2013	Child and maternal malnutrition	Other NCDs	0.30	0.00
2019	Child and maternal malnutrition	Other NCDs	0.24	0.00
2013	Childhood sexual abuse and bullying	Other NCDs	40.84	0.06
2019	Childhood sexual abuse and bullying	Other NCDs	40.56	0.06
2013	Diet high in processed meat	Cardiovascular diseases	66.55	3.24
2013	Diet high in processed meat	Neoplasms	43.12	2.06
2013	Diet high in processed meat	Diabetes mellitus	77.04	0.64
2019	Diet high in processed meat	Cardiovascular diseases	57.44	3.01
2019	Diet high in processed meat	Neoplasms	40.77	2.07
2019	Diet high in processed meat	Diabetes mellitus	85.26	0.64
2013	Diet high in red meat	Cardiovascular diseases	128.48	6.19
2013	Diet high in red meat	Neoplasms	29.10	1.41
2013	Diet high in red meat	Diabetes mellitus	27.51	0.26
2019	Diet high in red meat	Cardiovascular diseases	131.48	6.84
2019	Diet high in red meat	Neoplasms	30.18	1.58
2019	Diet high in red meat	Diabetes mellitus	33.54	0.29
2013	Diet high in sodium	Cardiovascular diseases	375.52	21.09
2013	Diet high in sodium	Neoplasms	61.66	3.61
2013	Diet high in sodium	Chronic kidney disease	50.90	2.71
2019	Diet high in sodium	Cardiovascular diseases	375.17	23.09
2019	Diet high in sodium	Neoplasms	57.49	3.66
2019	Diet high in sodium	Chronic kidney disease	50.64	3.05
2013	Diet high in sugar-sweetened beverages	Cardiovascular diseases	28.74	1.98
2013	Diet high in sugar-sweetened beverages	Diabetes mellitus	18.99	0.20
2019	Diet high in sugar-sweetened beverages	Cardiovascular diseases	29.99	2.24
2019	Diet high in sugar-sweetened beverages	Diabetes mellitus	24.23	0.23
2013	Diet high in trans fatty acids	Cardiovascular diseases	75.03	5.00
2019	Diet high in trans fatty acids	Cardiovascular diseases	75.05	5.47
2013	Diet low in calcium	Neoplasms	107.67	6.19
2019	Diet low in calcium	Neoplasms	108.78	6.76
2013	Diet low in fiber	Cardiovascular diseases	130.43	7.38
2013	Diet low in fiber	Neoplasms	15.99	0.93
2013	Diet low in fiber	Diabetes mellitus	17.10	0.19

2019	Diet low in fiber	Cardiovascular diseases	126.42	7.82
2019	Diet low in fiber	Neoplasms	16.04	1.01
2019	Diet low in fiber	Diabetes mellitus	19.81	0.20
2013	Diet low in fruits	Cardiovascular diseases	198.44	9.89
2013	Diet low in fruits	Neoplasms	76.97	4.22
2013	Diet low in fruits	Diabetes mellitus	38.32	0.39
2019	Diet low in fruits	Cardiovascular diseases	191.84	10.41
2019	Diet low in fruits	Neoplasms	71.56	4.22
2019	Diet low in fruits	Diabetes mellitus	42.95	0.40
2013	Diet low in legumes	Cardiovascular diseases	97.98	6.52
2019	Diet low in legumes	Cardiovascular diseases	92.35	6.86
2013	Diet low in milk	Neoplasms	134.70	7.55
2019	Diet low in milk	Neoplasms	139.71	8.45
2013	Diet low in nuts and seeds	Cardiovascular diseases	123.66	8.48
2013	Diet low in nuts and seeds	Diabetes mellitus	14.74	0.17
2019	Diet low in nuts and seeds	Cardiovascular diseases	123.05	9.23
2019	Diet low in nuts and seeds	Diabetes mellitus	17.48	0.18
2013	Diet low in polyunsaturated fatty acids	Cardiovascular diseases	38.50	2.50
2019	Diet low in polyunsaturated fatty acids	Cardiovascular diseases	37.88	2.69
2013	Diet low in seafood omega-3 fatty acids	Cardiovascular diseases	22.16	2.45
2019	Diet low in seafood omega-3 fatty acids	Cardiovascular diseases	43.71	4.11
2013	Diet low in vegetables	Cardiovascular diseases	35.81	2.29
2013	Diet low in vegetables	Neoplasms	3.27	0.18
2019	Diet low in vegetables	Cardiovascular diseases	35.59	2.51
2019	Diet low in vegetables	Neoplasms	3.21	0.19
2013	Diet low in whole grains	Cardiovascular diseases	245.91	15.10
2013	Diet low in whole grains	Neoplasms	119.84	6.65
2013	Diet low in whole grains	Diabetes mellitus	26.81	0.28
2019	Diet low in whole grains	Cardiovascular diseases	245.29	16.43
2019	Diet low in whole grains	Neoplasms	124.04	7.42
2019	Diet low in whole grains	Diabetes mellitus	31.69	0.31
2013	Drug use	Neoplasms	82.14	4.17
2013	Drug use	Other NCDs	176.61	3.25
2013	Drug use	Injurie	7.14	0.15
2019	Drug use	Neoplasms	87.56	4.74
2019	Drug use	Other NCDs	174.01	3.37
2019	Drug use	Injurie	6.07	0.13
2013	HPV infection	Neoplasms	NA	2.96
2019	HPV infection	Neoplasms	NA	3.42
2013	HTLV-1 infection	Neoplasms	NA	0.79
2019	HTLV-1 infection	Neoplasms	NA	0.72
2013	*Helicobacter pylori* infection	Neoplasms	NA	35.69
2019	*Helicobacter pylori* infection	Neoplasms	NA	38.57
2013	Hepatitis B virus infection	Neoplasms	55.49	2.63
2019	Hepatitis B virus infection	Neoplasms	52.85	2.64
2013	Hepatitis C virus infection	Neoplasms	301.89	18.46

2019	Hepatitis C virus infection	Neoplasms	300.20	19.60
2013	High LDL cholesterol	Cardiovascular diseases	846.99	53.05
2019	High LDL cholesterol	Cardiovascular diseases	859.49	59.30
2013	High body-mass index	Cardiovascular diseases	363.35	15.29
2013	High body-mass index	Neoplasms	157.99	8.40
2013	High body-mass index	Chronic kidney disease	89.97	3.89
2013	High body-mass index	Other NCDs	226.00	7.24
2013	High body-mass index	Chronic respiratory diseases	17.50	0.18
2013	High body-mass index	Diabetes mellitus	149.51	1.12
2019	High body-mass index	Cardiovascular diseases	365.35	16.45
2019	High body-mass index	Neoplasms	162.11	9.19
2019	High body-mass index	Chronic kidney disease	89.71	4.39
2019	High body-mass index	Other NCDs	251.62	9.14
2019	High body-mass index	Chronic respiratory diseases	18.35	0.20
2019	High body-mass index	Diabetes mellitus	178.55	1.18
2013	High fasting plasma glucose	Cardiovascular diseases	479.19	29.96
2013	High fasting plasma glucose	Neoplasms	167.35	10.16
2013	High fasting plasma glucose	Chronic kidney disease	241.42	12.43
2013	High fasting plasma glucose	Other NCDs	89.00	6.55
2013	High fasting plasma glucose	Diabetes mellitus	611.04	6.32
2019	High fasting plasma glucose	Cardiovascular diseases	561.22	37.29
2019	High fasting plasma glucose	Neoplasms	190.48	12.36
2019	High fasting plasma glucose	Chronic kidney disease	224.43	13.22
2019	High fasting plasma glucose	Other NCDs	119.83	9.15
2019	High fasting plasma glucose	Diabetes mellitus	717.75	6.87
2013	High systolic blood pressure	Cardiovascular diseases	2006.81	116.83
2013	High systolic blood pressure	Chronic kidney disease	349.20	19.66
2019	High systolic blood pressure	Cardiovascular diseases	2070.30	129.92
2019	High systolic blood pressure	Chronic kidney disease	373.48	23.76
2013	Intimate partner violence	Other NCDs	38.06	0.00
2013	Intimate partner violence	Injurie	6.01	0.05
2019	Intimate partner violence	Other NCDs	37.26	0.00
2019	Intimate partner violence	Injurie	6.12	0.05
2013	Kidney dysfunction	Cardiovascular diseases	337.36	22.39
2013	Kidney dysfunction	Chronic kidney disease	591.48	31.11
2013	Kidney dysfunction	Other NCDs	10.07	0.00
2019	Kidney dysfunction	Cardiovascular diseases	361.07	25.90
2019	Kidney dysfunction	Chronic kidney disease	601.61	35.47
2019	Kidney dysfunction	Other NCDs	11.98	0.00
2013	Low bone mineral density	Injurie	298.51	5.94
2019	Low bone mineral density	Injurie	348.71	6.98
2013	Low physical activity	Cardiovascular diseases	120.89	9.76
2013	Low physical activity	Neoplasms	49.52	3.27
2013	Low physical activity	Diabetes mellitus	52.17	0.71
2019	Low physical activity	Cardiovascular diseases	132.59	11.44
2019	Low physical activity	Neoplasms	54.09	3.83

2019	Low physical activity	Diabetes mellitus	63.84	0.80
2013	Tobacco smoking	Cardiovascular diseases	841.13	34.75
2013	Tobacco smoking	Neoplasms	1570.47	85.41
2013	Tobacco smoking	Other NCDs	604.11	13.01
2013	Tobacco smoking	Chronic respiratory diseases	360.14	14.31
2013	Tobacco smoking	Diabetes mellitus	106.69	0.86
2013	Tobacco smoking	Injurie	22.51	0.42
2019	Tobacco smoking	Cardiovascular diseases	743.56	31.81
2019	Tobacco smoking	Neoplasms	1445.03	83.75
2019	Tobacco smoking	Other NCDs	547.09	14.21
2019	Tobacco smoking	Chronic respiratory diseases	362.32	15.52
2019	Tobacco smoking	Diabetes mellitus	114.99	0.84
2019	Tobacco smoking	Injurie	22.10	0.40
2013	Unsafe sex	Neoplasms	79.46	3.22
2019	Unsafe sex	Neoplasms	76.26	3.29

* Data, except for hepatitis B and C virus infections, *Helicobacter pylori* infection, HPV infection, and HTLV-1 infection, were obtained from the GBD results tool: http://ghdx.healthdata.org/gbd-results-tool. DALYs: disability-adjusted life years; NCDs: non-communicable diseases; LDL: low-density lipoprotein; HPV: human papillomavirus; HTLV-1: human T-cell leukemia virus type 1. The order of the risk factors is sorted alphabetically.

Supplementary Table 4: All-age DALYs and number of deaths attributable to preventable behavioral and metabolic risk factors in 2013 and 2019 for women

Year	Risk factors	Cause	DALYs	Number of deaths
2013	Alcohol use	Cardiovascular diseases	-4769.32	-951.31
2013	Alcohol use	Neoplasms	104126.20	4695.02
2013	Alcohol use	Other NCDs	112780.66	4403.19
2013	Alcohol use	Diabetes mellitus	-21851.72	-229.54
2013	Alcohol use	Injurie	93029.66	1695.29
2019	Alcohol use	Cardiovascular diseases	-2555.15	-892.24
2019	Alcohol use	Neoplasms	104404.38	5066.85
2019	Alcohol use	Other NCDs	112936.65	4727.80
2019	Alcohol use	Diabetes mellitus	-26081.85	-246.76
2019	Alcohol use	Injurie	89108.32	1577.24
2013	Child and maternal malnutrition	Other NCDs	175.33	1.97
2019	Child and maternal malnutrition	Other NCDs	134.56	1.51
2013	Childhood sexual abuse and bullying	Other NCDs	27613.69	11.11
2019	Childhood sexual abuse and bullying	Other NCDs	27076.05	11.01
2013	Diet high in processed meat	Cardiovascular diseases	26072.47	1618.87
2013	Diet high in processed meat	Neoplasms	22749.92	1173.30
2013	Diet high in processed meat	Diabetes mellitus	41019.68	370.72
2019	Diet high in processed meat	Cardiovascular diseases	23199.27	1570.05
2019	Diet high in processed meat	Neoplasms	21405.61	1184.09
2019	Diet high in processed meat	Diabetes mellitus	46851.95	376.05
2013	Diet high in red meat	Cardiovascular diseases	67651.83	3780.15
2013	Diet high in red meat	Neoplasms	21425.00	1041.65
2013	Diet high in red meat	Diabetes mellitus	14418.11	163.88
2019	Diet high in red meat	Cardiovascular diseases	70846.44	4270.82
2019	Diet high in red meat	Neoplasms	21799.19	1146.16
2019	Diet high in red meat	Diabetes mellitus	18183.91	184.30
2013	Diet high in sodium	Cardiovascular diseases	172352.42	11894.31
2013	Diet high in sodium	Neoplasms	24162.12	1597.90
2013	Diet high in sodium	Chronic kidney disease	25665.56	1576.59
2019	Diet high in sodium	Cardiovascular diseases	178760.23	13439.42
2019	Diet high in sodium	Neoplasms	22757.14	1643.12
2019	Diet high in sodium	Chronic kidney disease	25710.71	1796.04
2013	Diet high in sugar-sweetened beverages	Cardiovascular diseases	13281.14	1214.41
2013	Diet high in sugar-sweetened beverages	Diabetes mellitus	10321.05	129.15
2019	Diet high in sugar-sweetened beverages	Cardiovascular diseases	14344.62	1388.49
2019	Diet high in sugar-sweetened beverages	Diabetes mellitus	13656.64	149.72
2013	Diet high in trans fatty acids	Cardiovascular diseases	36701.42	3208.21
2019	Diet high in trans fatty acids	Cardiovascular diseases	38052.25	3571.02
2013	Diet low in calcium	Neoplasms	58052.58	3869.23
2019	Diet low in calcium	Neoplasms	58857.39	4247.07
2013	Diet low in fiber	Cardiovascular diseases	74716.44	5189.55
2013	Diet low in fiber	Neoplasms	9601.15	648.91
2013	Diet low in fiber	Diabetes mellitus	9637.87	133.05

2019	Diet low in fiber	Cardiovascular diseases	75233.11	5613.02
2019	Diet low in fiber	Neoplasms	9768.26	713.00
2019	Diet low in fiber	Diabetes mellitus	11618.38	143.80
2013	Diet low in fruits	Cardiovascular diseases	102913.06	6135.46
2013	Diet low in fruits	Neoplasms	21201.93	1351.68
2013	Diet low in fruits	Diabetes mellitus	19245.44	244.16
2019	Diet low in fruits	Cardiovascular diseases	102950.30	6685.46
2019	Diet low in fruits	Neoplasms	21252.98	1465.23
2019	Diet low in fruits	Diabetes mellitus	22598.41	260.78
2013	Diet low in legumes	Cardiovascular diseases	49730.73	4460.60
2019	Diet low in legumes	Cardiovascular diseases	49194.60	4802.56
2013	Diet low in milk	Neoplasms	72510.68	4634.63
2019	Diet low in milk	Neoplasms	75290.08	5211.05
2013	Diet low in nuts and seeds	Cardiovascular diseases	58184.57	5319.32
2013	Diet low in nuts and seeds	Diabetes mellitus	7925.82	111.13
2019	Diet low in nuts and seeds	Cardiovascular diseases	60177.07	5877.65
2019	Diet low in nuts and seeds	Diabetes mellitus	9694.22	122.11
2013	Diet low in polyunsaturated fatty acids	Cardiovascular diseases	18348.59	1580.87
2019	Diet low in polyunsaturated fatty acids	Cardiovascular diseases	18762.30	1737.12
2013	Diet low in seafood omega-3 fatty acids	Cardiovascular diseases	15821.57	2114.26
2019	Diet low in seafood omega-3 fatty acids	Cardiovascular diseases	25805.90	3086.75
2013	Diet low in vegetables	Cardiovascular diseases	21098.71	1669.69
2013	Diet low in vegetables	Neoplasms	733.32	49.28
2019	Diet low in vegetables	Cardiovascular diseases	21409.35	1828.72
2019	Diet low in vegetables	Neoplasms	728.64	52.78
2013	Diet low in whole grains	Cardiovascular diseases	113647.62	9011.11
2013	Diet low in whole grains	Neoplasms	63247.92	3989.41
2013	Diet low in whole grains	Diabetes mellitus	14143.09	176.88
2019	Diet low in whole grains	Cardiovascular diseases	117905.69	10043.34
2019	Diet low in whole grains	Neoplasms	65471.58	4471.18
2019	Diet low in whole grains	Diabetes mellitus	17243.72	194.20
2013	Drug use	Neoplasms	36639.44	2121.69
2013	Drug use	Other NCDs	92926.65	1696.06
2013	Drug use	Injurie	2261.04	47.05
2019	Drug use	Neoplasms	39766.98	2457.88
2019	Drug use	Other NCDs	90061.42	1769.50
2019	Drug use	Injurie	1854.02	39.78
2013	HPV infection	Neoplasms	NA	3170.19
2019	HPV infection	Neoplasms	NA	3547.83
2013	HTLV-1 infection	Neoplasms	NA	500.00
2019	HTLV-1 infection	Neoplasms	NA	430.00
2013	*Helicobacter pylori* infection	Neoplasms	NA	16080.88
2019	*Helicobacter pylori* infection	Neoplasms	NA	17493.30
2013	Hepatitis B virus infection	Neoplasms	16456.26	939.36
2019	Hepatitis B virus infection	Neoplasms	15883.52	960.88
2013	Hepatitis C virus infection	Neoplasms	121794.17	8784.91

2019	Hepatitis C virus infection	Neoplasms	122770.70	9400.62
2013	High LDL cholesterol	Cardiovascular diseases	442578.66	35295.86
2019	High LDL cholesterol	Cardiovascular diseases	467099.81	40177.75
2013	High body-mass index	Cardiovascular diseases	184854.56	9076.57
2013	High body-mass index	Neoplasms	79511.45	4682.84
2013	High body-mass index	Chronic kidney disease	54025.33	2584.80
2013	High body-mass index	Other NCDs	189904.13	6757.23
2013	High body-mass index	Chronic respiratory diseases	13432.56	137.36
2013	High body-mass index	Diabetes mellitus	77803.29	652.20
2019	High body-mass index	Cardiovascular diseases	193024.03	10210.92
2019	High body-mass index	Neoplasms	82683.59	5171.16
2019	High body-mass index	Chronic kidney disease	54663.70	2987.19
2019	High body-mass index	Other NCDs	210213.13	8306.55
2019	High body-mass index	Chronic respiratory diseases	14195.61	153.14
2019	High body-mass index	Diabetes mellitus	97506.52	714.09
2013	High fasting plasma glucose	Cardiovascular diseases	206333.84	15537.50
2013	High fasting plasma glucose	Neoplasms	70258.66	4597.75
2013	High fasting plasma glucose	Chronic kidney disease	146868.06	8454.00
2013	High fasting plasma glucose	Other NCDs	67826.74	5296.02
2013	High fasting plasma glucose	Diabetes mellitus	331745.62	4032.23
2019	High fasting plasma glucose	Cardiovascular diseases	253892.22	20034.62
2019	High fasting plasma glucose	Neoplasms	85577.94	5972.73
2019	High fasting plasma glucose	Chronic kidney disease	133058.77	8969.64
2019	High fasting plasma glucose	Other NCDs	91342.85	7306.93
2019	High fasting plasma glucose	Diabetes mellitus	400667.81	4436.76
2013	High systolic blood pressure	Cardiovascular diseases	1072398.70	74519.96
2013	High systolic blood pressure	Chronic kidney disease	202915.34	12879.54
2019	High systolic blood pressure	Cardiovascular diseases	1135872.16	84592.67
2019	High systolic blood pressure	Chronic kidney disease	219653.83	15880.24
2013	Intimate partner violence	Other NCDs	49350.63	0.00
2013	Intimate partner violence	Injurie	7787.61	71.09
2019	Intimate partner violence	Other NCDs	47613.55	0.00
2019	Intimate partner violence	Injurie	7821.76	66.85
2013	Kidney dysfunction	Cardiovascular diseases	171760.84	13919.89
2013	Kidney dysfunction	Chronic kidney disease	357991.34	20963.93
2013	Kidney dysfunction	Other NCDs	3906.20	0.00
2019	Kidney dysfunction	Cardiovascular diseases	185282.11	16022.11
2019	Kidney dysfunction	Chronic kidney disease	364228.41	23980.87
2019	Kidney dysfunction	Other NCDs	4541.26	0.00
2013	Low bone mineral density	Injurie	221738.25	3705.97
2019	Low bone mineral density	Injurie	259854.33	4325.56
2013	Low physical activity	Cardiovascular diseases	84440.44	7827.56
2013	Low physical activity	Neoplasms	35100.27	2491.20
2013	Low physical activity	Diabetes mellitus	33759.13	536.46
2019	Low physical activity	Cardiovascular diseases	94455.45	9208.77
2019	Low physical activity	Neoplasms	38026.79	2902.39

2019	Low physical activity	Diabetes mellitus	42207.89	607.94
2013	Tobacco smoking	Cardiovascular diseases	240289.70	10006.64
2013	Tobacco smoking	Neoplasms	346521.88	19107.71
2013	Tobacco smoking	Other NCDs	276101.67	5060.88
2013	Tobacco smoking	Chronic respiratory diseases	90428.28	3429.07
2013	Tobacco smoking	Diabetes mellitus	36786.26	331.68
2013	Tobacco smoking	Injurie	8072.94	86.11
2019	Tobacco smoking	Cardiovascular diseases	223367.14	9789.15
2019	Tobacco smoking	Neoplasms	332794.34	19646.64
2019	Tobacco smoking	Other NCDs	247413.32	5434.25
2019	Tobacco smoking	Chronic respiratory diseases	90781.95	3671.75
2019	Tobacco smoking	Diabetes mellitus	43332.55	349.79
2019	Tobacco smoking	Injurie	8393.80	86.42
2013	Unsafe sex	Neoplasms	103031.44	4172.02
2019	Unsafe sex	Neoplasms	97453.56	4210.49

* Data, except for hepatitis B and C virus infections, *Helicobacter pylori* infection, HPV infection, and HTLV-1 infection, were obtained from the GBD results tool: http://ghdx.healthdata.org/gbd-results-tool. DALYs: disability-adjusted life years; NCDs: non-communicable diseases; LDL: low-density lipoprotein; HPV: human papillomavirus; HTLV-1: human T-cell leukemia virus type 1. The order of the risk factors is sorted alphabetically.

Supplementary Table 5: All-age DALYs rate (per 100,000) and mortality rate (per 100,000) attributable to preventable behavioral and metabolic risk factors in 2013 and 2019 for women

Year	Risk factors	Cause	DALYs rate	Mortality rate
2013	Alcohol use	Cardiovascular diseases	-7.18	-1.43
2013	Alcohol use	Neoplasms	156.69	7.07
2013	Alcohol use	Other NCDs	169.72	6.63
2013	Alcohol use	Diabetes mellitus	-32.88	-0.35
2013	Alcohol use	Injurie	139.99	2.55
2019	Alcohol use	Cardiovascular diseases	-3.90	-1.36
2019	Alcohol use	Neoplasms	159.35	7.73
2019	Alcohol use	Other NCDs	172.38	7.22
2019	Alcohol use	Diabetes mellitus	-39.81	-0.38
2019	Alcohol use	Injurie	136.01	2.41
2013	Child and maternal malnutrition	Other NCDs	0.26	0.00
2019	Child and maternal malnutrition	Other NCDs	0.21	0.00
2013	Childhood sexual abuse and bullying	Other NCDs	41.55	0.02
2019	Childhood sexual abuse and bullying	Other NCDs	41.33	0.02
2013	Diet high in processed meat	Cardiovascular diseases	39.23	2.44
2013	Diet high in processed meat	Neoplasms	34.23	1.77
2013	Diet high in processed meat	Diabetes mellitus	61.73	0.56
2019	Diet high in processed meat	Cardiovascular diseases	35.41	2.40
2019	Diet high in processed meat	Neoplasms	32.67	1.81
2019	Diet high in processed meat	Diabetes mellitus	71.51	0.57
2013	Diet high in red meat	Cardiovascular diseases	101.80	5.69
2013	Diet high in red meat	Neoplasms	32.24	1.57
2013	Diet high in red meat	Diabetes mellitus	21.70	0.25
2019	Diet high in red meat	Cardiovascular diseases	108.13	6.52
2019	Diet high in red meat	Neoplasms	33.27	1.75
2019	Diet high in red meat	Diabetes mellitus	27.75	0.28
2013	Diet high in sodium	Cardiovascular diseases	259.36	17.90
2013	Diet high in sodium	Neoplasms	36.36	2.40
2013	Diet high in sodium	Chronic kidney disease	38.62	2.37
2019	Diet high in sodium	Cardiovascular diseases	272.84	20.51
2019	Diet high in sodium	Neoplasms	34.73	2.51
2019	Diet high in sodium	Chronic kidney disease	39.24	2.74
2013	Diet high in sugar-sweetened beverages	Cardiovascular diseases	19.99	1.83
2013	Diet high in sugar-sweetened beverages	Diabetes mellitus	15.53	0.19
2019	Diet high in sugar-sweetened beverages	Cardiovascular diseases	21.89	2.12
2019	Diet high in sugar-sweetened beverages	Diabetes mellitus	20.84	0.23
2013	Diet high in trans fatty acids	Cardiovascular diseases	55.23	4.83
2019	Diet high in trans fatty acids	Cardiovascular diseases	58.08	5.45
2013	Diet low in calcium	Neoplasms	87.36	5.82
2019	Diet low in calcium	Neoplasms	89.83	6.48
2013	Diet low in fiber	Cardiovascular diseases	112.44	7.81
2013	Diet low in fiber	Neoplasms	14.45	0.98
2013	Diet low in fiber	Diabetes mellitus	14.50	0.20

2019	Diet low in fiber	Cardiovascular diseases	114.83	8.57
2019	Diet low in fiber	Neoplasms	14.91	1.09
2019	Diet low in fiber	Diabetes mellitus	17.73	0.22
2013	Diet low in fruits	Cardiovascular diseases	154.87	9.23
2013	Diet low in fruits	Neoplasms	31.91	2.03
2013	Diet low in fruits	Diabetes mellitus	28.96	0.37
2019	Diet low in fruits	Cardiovascular diseases	157.13	10.20
2019	Diet low in fruits	Neoplasms	32.44	2.24
2019	Diet low in fruits	Diabetes mellitus	34.49	0.40
2013	Diet low in legumes	Cardiovascular diseases	74.84	6.71
2019	Diet low in legumes	Cardiovascular diseases	75.09	7.33
2013	Diet low in milk	Neoplasms	109.12	6.97
2019	Diet low in milk	Neoplasms	114.92	7.95
2013	Diet low in nuts and seeds	Cardiovascular diseases	87.56	8.00
2013	Diet low in nuts and seeds	Diabetes mellitus	11.93	0.17
2019	Diet low in nuts and seeds	Cardiovascular diseases	91.85	8.97
2019	Diet low in nuts and seeds	Diabetes mellitus	14.80	0.19
2013	Diet low in polyunsaturated fatty acids	Cardiovascular diseases	27.61	2.38
2019	Diet low in polyunsaturated fatty acids	Cardiovascular diseases	28.64	2.65
2013	Diet low in seafood omega-3 fatty acids	Cardiovascular diseases	23.81	3.18
2019	Diet low in seafood omega-3 fatty acids	Cardiovascular diseases	39.39	4.71
2013	Diet low in vegetables	Cardiovascular diseases	31.75	2.51
2013	Diet low in vegetables	Neoplasms	1.10	0.07
2019	Diet low in vegetables	Cardiovascular diseases	32.68	2.79
2019	Diet low in vegetables	Neoplasms	1.11	0.08
2013	Diet low in whole grains	Cardiovascular diseases	171.02	13.56
2013	Diet low in whole grains	Neoplasms	95.18	6.00
2013	Diet low in whole grains	Diabetes mellitus	21.28	0.27
2019	Diet low in whole grains	Cardiovascular diseases	179.96	15.33
2019	Diet low in whole grains	Neoplasms	99.93	6.82
2019	Diet low in whole grains	Diabetes mellitus	26.32	0.30
2013	Drug use	Neoplasms	55.14	3.19
2013	Drug use	Other NCDs	139.84	2.55
2013	Drug use	Injurie	3.40	0.07
2019	Drug use	Neoplasms	60.70	3.75
2019	Drug use	Other NCDs	137.46	2.70
2019	Drug use	Injurie	2.83	0.06
2013	HPV infection	Neoplasms	NA	4.77
2019	HPV infection	Neoplasms	NA	5.42
2013	HTLV-1 infection	Neoplasms	NA	0.75
2019	HTLV-1 infection	Neoplasms	NA	0.66
2013	*Helicobacter pylori* infection	Neoplasms	NA	24.20
2019	*Helicobacter pylori* infection	Neoplasms	NA	26.70
2013	Hepatitis B virus infection	Neoplasms	24.76	1.41
2019	Hepatitis B virus infection	Neoplasms	24.24	1.47
2013	Hepatitis C virus infection	Neoplasms	183.28	13.22

2019	Hepatitis C virus infection	Neoplasms	187.38	14.35
2013	High LDL cholesterol	Cardiovascular diseases	666.01	53.11
2019	High LDL cholesterol	Cardiovascular diseases	712.93	61.32
2013	High body-mass index	Cardiovascular diseases	278.18	13.66
2013	High body-mass index	Neoplasms	119.65	7.05
2013	High body-mass index	Chronic kidney disease	81.30	3.89
2013	High body-mass index	Other NCDs	285.77	10.17
2013	High body-mass index	Chronic respiratory diseases	20.21	0.21
2013	High body-mass index	Diabetes mellitus	117.08	0.98
2019	High body-mass index	Cardiovascular diseases	294.61	15.58
2019	High body-mass index	Neoplasms	126.20	7.89
2019	High body-mass index	Chronic kidney disease	83.43	4.56
2019	High body-mass index	Other NCDs	320.85	12.68
2019	High body-mass index	Chronic respiratory diseases	21.67	0.23
2019	High body-mass index	Diabetes mellitus	148.82	1.09
2013	High fasting plasma glucose	Cardiovascular diseases	310.50	23.38
2013	High fasting plasma glucose	Neoplasms	105.73	6.92
2013	High fasting plasma glucose	Chronic kidney disease	221.01	12.72
2013	High fasting plasma glucose	Other NCDs	102.07	7.97
2013	High fasting plasma glucose	Diabetes mellitus	499.22	6.07
2019	High fasting plasma glucose	Cardiovascular diseases	387.52	30.58
2019	High fasting plasma glucose	Neoplasms	130.62	9.12
2019	High fasting plasma glucose	Chronic kidney disease	203.09	13.69
2019	High fasting plasma glucose	Other NCDs	139.42	11.15
2019	High fasting plasma glucose	Diabetes mellitus	611.54	6.77
2013	High systolic blood pressure	Cardiovascular diseases	1613.78	112.14
2013	High systolic blood pressure	Chronic kidney disease	305.35	19.38
2019	High systolic blood pressure	Cardiovascular diseases	1733.68	129.11
2019	High systolic blood pressure	Chronic kidney disease	335.26	24.24
2013	Intimate partner violence	Other NCDs	74.26	0.00
2013	Intimate partner violence	Injurie	11.72	0.11
2019	Intimate partner violence	Other NCDs	72.67	0.00
2019	Intimate partner violence	Injurie	11.94	0.10
2013	Kidney dysfunction	Cardiovascular diseases	258.47	20.95
2013	Kidney dysfunction	Chronic kidney disease	538.72	31.55
2013	Kidney dysfunction	Other NCDs	5.88	0.00
2019	Kidney dysfunction	Cardiovascular diseases	282.80	24.45
2019	Kidney dysfunction	Chronic kidney disease	555.92	36.60
2019	Kidney dysfunction	Other NCDs	6.93	0.00
2013	Low bone mineral density	Injurie	333.68	5.58
2019	Low bone mineral density	Injurie	396.62	6.60
2013	Low physical activity	Cardiovascular diseases	127.07	11.78
2013	Low physical activity	Neoplasms	52.82	3.75
2013	Low physical activity	Diabetes mellitus	50.80	0.81
2019	Low physical activity	Cardiovascular diseases	144.17	14.06
2019	Low physical activity	Neoplasms	58.04	4.43

2019	Low physical activity	Diabetes mellitus	64.42	0.93
2013	Tobacco smoking	Cardiovascular diseases	361.60	15.06
2013	Tobacco smoking	Neoplasms	521.46	28.75
2013	Tobacco smoking	Other NCDs	415.49	7.62
2013	Tobacco smoking	Chronic respiratory diseases	136.08	5.16
2013	Tobacco smoking	Diabetes mellitus	55.36	0.50
2013	Tobacco smoking	Injurie	12.15	0.13
2019	Tobacco smoking	Cardiovascular diseases	340.93	14.94
2019	Tobacco smoking	Neoplasms	507.94	29.99
2019	Tobacco smoking	Other NCDs	377.63	8.29
2019	Tobacco smoking	Chronic respiratory diseases	138.56	5.60
2019	Tobacco smoking	Diabetes mellitus	66.14	0.53
2019	Tobacco smoking	Injurie	12.81	0.13
2013	Unsafe sex	Neoplasms	155.05	6.28
2019	Unsafe sex	Neoplasms	148.74	6.43

* Data, except for hepatitis B and C virus infections, *Helicobacter pylori* infection, HPV infection, and HTLV-1 infection, were obtained from the GBD results tool: http://ghdx.healthdata.org/gbd-results-tool. DALYs: disability-adjusted life years; NCDs: non-communicable diseases; LDL: low-density lipoprotein; HPV: human papillomavirus; HTLV-1: human T-cell leukemia virus type 1. The order of the risk factors is sorted alphabetically.

Supplementary Table 6: All-age DALYs and number of deaths attributable to preventable behavioral and metabolic risk factors in 2013 and 2019 for men

Year	Risk factors	Cause	DALYs	Number of deaths
2013	Alcohol use	Cardiovascular diseases	63700.12	1699.26
2013	Alcohol use	Neoplasms	268790.69	12682.94
2013	Alcohol use	Other NCDs	326908.39	10581.95
2013	Alcohol use	Diabetes mellitus	3153.03	8.04
2013	Alcohol use	Injurie	271511.62	6061.16
2019	Alcohol use	Cardiovascular diseases	70002.31	2078.16
2019	Alcohol use	Neoplasms	264253.56	13218.62
2019	Alcohol use	Other NCDs	312618.75	10516.32
2019	Alcohol use	Diabetes mellitus	4429.45	11.99
2019	Alcohol use	Injurie	245454.50	5496.29
2013	Child and maternal malnutrition	Other NCDs	214.22	2.41
2019	Child and maternal malnutrition	Other NCDs	169.12	1.90
2013	Childhood sexual abuse and bullying	Other NCDs	25335.82	63.75
2019	Childhood sexual abuse and bullying	Other NCDs	24758.73	62.59
2013	Diet high in processed meat	Cardiovascular diseases	60218.16	2588.32
2013	Diet high in processed meat	Neoplasms	33155.99	1498.50
2013	Diet high in processed meat	Diabetes mellitus	58868.98	464.09
2019	Diet high in processed meat	Cardiovascular diseases	50202.43	2275.12
2019	Diet high in processed meat	Neoplasms	30690.57	1455.28
2019	Diet high in processed meat	Diabetes mellitus	62095.56	439.19
2013	Diet high in red meat	Cardiovascular diseases	98928.25	4244.76
2013	Diet high in red meat	Neoplasms	16301.12	791.27
2013	Diet high in red meat	Diabetes mellitus	21247.59	178.38
2019	Diet high in red meat	Cardiovascular diseases	97173.11	4474.90
2019	Diet high in red meat	Neoplasms	16766.92	869.39
2019	Diet high in red meat	Diabetes mellitus	24678.21	190.54
2013	Diet high in sodium	Cardiovascular diseases	314531.62	15454.09
2013	Diet high in sodium	Neoplasms	55781.70	3086.28
2013	Diet high in sodium	Chronic kidney disease	40327.21	1934.57
2019	Diet high in sodium	Cardiovascular diseases	300667.66	16067.21
2019	Diet high in sodium	Neoplasms	50707.55	3034.29
2019	Diet high in sodium	Chronic kidney disease	39005.58	2107.41
2013	Diet high in sugar-sweetened beverages	Cardiovascular diseases	23978.15	1357.32
2013	Diet high in sugar-sweetened beverages	Diabetes mellitus	14305.43	126.88
2019	Diet high in sugar-sweetened beverages	Cardiovascular diseases	23983.50	1469.04
2019	Diet high in sugar-sweetened beverages	Diabetes mellitus	17302.43	140.72
2013	Diet high in trans fatty acids	Cardiovascular diseases	60582.08	3271.96
2019	Diet high in trans fatty acids	Cardiovascular diseases	57859.25	3413.19
2013	Diet low in calcium	Neoplasms	81551.26	4158.46
2019	Diet low in calcium	Neoplasms	80146.58	4389.01
2013	Diet low in fiber	Cardiovascular diseases	94395.79	4378.25
2013	Diet low in fiber	Neoplasms	11133.99	559.60
2013	Diet low in fiber	Diabetes mellitus	12536.98	113.25

2019	Diet low in fiber	Cardiovascular diseases	86320.36	4376.69
2019	Diet low in fiber	Neoplasms	10731.77	583.40
2019	Diet low in fiber	Diabetes mellitus	13701.23	115.11
2013	Diet low in fruits	Cardiovascular diseases	154375.75	6685.48
2013	Diet low in fruits	Neoplasms	78597.97	4120.18
2013	Diet low in fruits	Diabetes mellitus	30442.35	255.57
2019	Diet low in fruits	Cardiovascular diseases	142203.95	6620.10
2019	Diet low in fruits	Neoplasms	70198.67	3926.59
2019	Diet low in fruits	Diabetes mellitus	32288.14	247.72
2013	Diet low in legumes	Cardiovascular diseases	77300.90	3986.88
2019	Diet low in legumes	Cardiovascular diseases	68823.31	3960.73
2013	Diet low in milk	Neoplasms	102143.55	5153.29
2019	Diet low in milk	Neoplasms	103243.38	5582.83
2013	Diet low in nuts and seeds	Cardiovascular diseases	102149.87	5677.37
2013	Diet low in nuts and seeds	Diabetes mellitus	11185.93	108.04
2019	Diet low in nuts and seeds	Cardiovascular diseases	97060.55	5917.17
2019	Diet low in nuts and seeds	Diabetes mellitus	12645.78	113.75
2013	Diet low in polyunsaturated fatty acids	Cardiovascular diseases	31573.06	1665.90
2019	Diet low in polyunsaturated fatty acids	Cardiovascular diseases	29643.79	1705.70
2013	Diet low in seafood omega-3 fatty acids	Cardiovascular diseases	12906.09	1066.96
2019	Diet low in seafood omega-3 fatty acids	Cardiovascular diseases	30048.12	2170.80
2013	Diet low in vegetables	Cardiovascular diseases	25327.72	1297.31
2013	Diet low in vegetables	Neoplasms	3501.92	180.75
2019	Diet low in vegetables	Cardiovascular diseases	24075.64	1378.56
2019	Diet low in vegetables	Neoplasms	3379.06	187.73
2013	Diet low in whole grains	Cardiovascular diseases	205195.92	10560.91
2013	Diet low in whole grains	Neoplasms	92137.35	4636.55
2013	Diet low in whole grains	Diabetes mellitus	20621.01	187.35
2019	Diet low in whole grains	Cardiovascular diseases	195550.64	10956.18
2019	Diet low in whole grains	Neoplasms	93036.16	5016.62
2019	Diet low in whole grains	Diabetes mellitus	23254.13	195.77
2013	Drug use	Neoplasms	69861.87	3290.17
2013	Drug use	Other NCDs	136067.03	2520.87
2013	Drug use	Injurie	6997.46	142.66
2019	Drug use	Neoplasms	72121.75	3599.32
2019	Drug use	Other NCDs	132297.62	2541.16
2019	Drug use	Injurie	5908.68	122.01
2013	HPV infection	Neoplasms	NA	662.63
2019	HPV infection	Neoplasms	NA	818.70
2013	HTLV-1 infection	Neoplasms	NA	528.00
2019	HTLV-1 infection	Neoplasms	NA	489.00
2013	*Helicobacter pylori* infection	Neoplasms	NA	30191.91
2019	*Helicobacter pylori* infection	Neoplasms	NA	31790.48
2013	Hepatitis B virus infection	Neoplasms	55494.00	2467.75
2019	Hepatitis B virus infection	Neoplasms	51654.98	2407.27
2013	Hepatitis C virus infection	Neoplasms	269625.97	15145.71

2019	Hepatitis C virus infection	Neoplasms	260854.47	15650.88
2013	High LDL cholesterol	Cardiovascular diseases	655602.19	33492.92
2019	High LDL cholesterol	Cardiovascular diseases	631235.06	35604.28
2013	High body-mass index	Cardiovascular diseases	286255.34	10749.48
2013	High body-mass index	Neoplasms	125327.31	6211.20
2013	High body-mass index	Chronic kidney disease	62622.12	2458.52
2013	High body-mass index	Other NCDs	103124.61	2635.19
2013	High body-mass index	Chronic respiratory diseases	9258.00	94.80
2013	High body-mass index	Diabetes mellitus	116047.09	793.78
2019	High body-mass index	Cardiovascular diseases	273850.34	10813.48
2019	High body-mass index	Neoplasms	124470.50	6569.80
2019	High body-mass index	Chronic kidney disease	59969.98	2627.82
2019	High body-mass index	Other NCDs	111328.10	3374.51
2019	High body-mass index	Chronic respiratory diseases	9257.14	97.16
2019	High body-mass index	Diabetes mellitus	130656.66	796.27
2013	High fasting plasma glucose	Cardiovascular diseases	414972.56	23310.86
2013	High fasting plasma glucose	Neoplasms	146719.70	8573.69
2013	High fasting plasma glucose	Chronic kidney disease	166151.47	7656.77
2013	High fasting plasma glucose	Other NCDs	47568.02	3201.72
2013	High fasting plasma glucose	Diabetes mellitus	460516.78	4157.82
2019	High fasting plasma glucose	Cardiovascular diseases	463280.16	27611.83
2019	High fasting plasma glucose	Neoplasms	157831.97	9821.23
2019	High fasting plasma glucose	Chronic kidney disease	153738.03	7923.12
2019	High fasting plasma glucose	Other NCDs	61790.84	4384.66
2019	High fasting plasma glucose	Diabetes mellitus	516539.47	4347.33
2013	High systolic blood pressure	Cardiovascular diseases	1529577.12	76958.17
2013	High systolic blood pressure	Chronic kidney disease	249841.53	12605.33
2019	High systolic blood pressure	Cardiovascular diseases	1509737.27	81431.28
2019	High systolic blood pressure	Chronic kidney disease	257607.06	14480.42
2013	Intimate partner violence	Other NCDs	0.00	0.00
2013	Intimate partner violence	Injurie	0.00	0.00
2019	Intimate partner violence	Other NCDs	0.00	0.00
2019	Intimate partner violence	Injurie	0.00	0.00
2013	Kidney dysfunction	Cardiovascular diseases	265645.00	15110.68
2013	Kidney dysfunction	Chronic kidney disease	408908.25	19376.14
2013	Kidney dysfunction	Other NCDs	9144.09	0.00
2019	Kidney dysfunction	Cardiovascular diseases	276119.31	17071.19
2019	Kidney dysfunction	Chronic kidney disease	404564.91	21343.56
2019	Kidney dysfunction	Other NCDs	10764.45	0.00
2013	Low bone mineral density	Injurie	165297.06	3989.82
2019	Low bone mineral density	Injurie	185757.64	4595.22
2013	Low physical activity	Cardiovascular diseases	72302.50	4822.24
2013	Low physical activity	Neoplasms	29109.94	1751.81
2013	Low physical activity	Diabetes mellitus	33880.21	380.85
2019	Low physical activity	Cardiovascular diseases	74974.64	5406.39
2019	Low physical activity	Neoplasms	31087.91	1994.13

2019	Low physical activity	Diabetes mellitus	39369.64	416.69
2013	Tobacco smoking	Cardiovascular diseases	850292.94	35045.30
2013	Tobacco smoking	Neoplasms	1689700.37	91627.79
2013	Tobacco smoking	Other NCDs	507170.05	11812.18
2013	Tobacco smoking	Chronic respiratory diseases	376513.94	15126.28
2013	Tobacco smoking	Diabetes mellitus	101551.32	782.83
2013	Tobacco smoking	Injurie	21117.97	455.89
2019	Tobacco smoking	Cardiovascular diseases	726817.50	30861.47
2019	Tobacco smoking	Neoplasms	1513789.52	87373.25
2019	Tobacco smoking	Other NCDs	451702.54	12719.16
2019	Tobacco smoking	Chronic respiratory diseases	372226.25	16161.80
2019	Tobacco smoking	Diabetes mellitus	103605.60	719.46
2019	Tobacco smoking	Injurie	19852.30	424.75
2013	Unsafe sex	Neoplasms	0.00	0.00
2019	Unsafe sex	Neoplasms	0.00	0.00

* Data, except for hepatitis B and C virus infections, *Helicobacter pylori* infection, HPV infection, and HTLV-1 infection, were obtained from the GBD results tool: http://ghdx.healthdata.org/gbd-results-tool. DALYs: disability-adjusted life years; NCDs: non-communicable diseases; LDL: low-density lipoprotein; HPV: human papillomavirus; HTLV-1: human T-cell leukemia virus type 1. The order of the risk factors is sorted alphabetically.

Supplementary Table 7: All-age DALYs rate (per 100,000) and mortality rate (per 100,000) attributable to preventable behavioral and metabolic risk factors in 2013 and 2019 for men

Year	Risk factors	Cause	DALYs rate	Mortality rate
2013	Alcohol use	Cardiovascular diseases	100.78	2.69
2013	Alcohol use	Neoplasms	425.27	20.07
2013	Alcohol use	Other NCDs	517.22	16.74
2013	Alcohol use	Diabetes mellitus	4.99	0.01
2013	Alcohol use	Injurie	429.58	9.59
2019	Alcohol use	Cardiovascular diseases	112.42	3.34
2019	Alcohol use	Neoplasms	424.36	21.23
2019	Alcohol use	Other NCDs	502.03	16.89
2019	Alcohol use	Diabetes mellitus	7.11	0.02
2019	Alcohol use	Injurie	394.17	8.83
2013	Child and maternal malnutrition	Other NCDs	0.34	0.00
2019	Child and maternal malnutrition	Other NCDs	0.27	0.00
2013	Childhood sexual abuse and bullying	Other NCDs	40.09	0.10
2019	Childhood sexual abuse and bullying	Other NCDs	39.76	0.10
2013	Diet high in processed meat	Cardiovascular diseases	95.27	4.10
2013	Diet high in processed meat	Neoplasms	52.46	2.37
2013	Diet high in processed meat	Diabetes mellitus	93.14	0.73
2019	Diet high in processed meat	Cardiovascular diseases	80.62	3.65
2019	Diet high in processed meat	Neoplasms	49.29	2.34
2019	Diet high in processed meat	Diabetes mellitus	99.72	0.71
2013	Diet high in red meat	Cardiovascular diseases	156.52	6.72
2013	Diet high in red meat	Neoplasms	25.79	1.25
2013	Diet high in red meat	Diabetes mellitus	33.62	0.28
2019	Diet high in red meat	Cardiovascular diseases	156.05	7.19
2019	Diet high in red meat	Neoplasms	26.93	1.40
2019	Diet high in red meat	Diabetes mellitus	39.63	0.31
2013	Diet high in sodium	Cardiovascular diseases	497.64	24.45
2013	Diet high in sodium	Neoplasms	88.26	4.88
2013	Diet high in sodium	Chronic kidney disease	63.80	3.06
2019	Diet high in sodium	Cardiovascular diseases	482.84	25.80
2019	Diet high in sodium	Neoplasms	81.43	4.87
2019	Diet high in sodium	Chronic kidney disease	62.64	3.38
2013	Diet high in sugar-sweetened beverages	Cardiovascular diseases	37.94	2.15
2013	Diet high in sugar-sweetened beverages	Diabetes mellitus	22.63	0.20
2019	Diet high in sugar-sweetened beverages	Cardiovascular diseases	38.52	2.36
2019	Diet high in sugar-sweetened beverages	Diabetes mellitus	27.79	0.23
2013	Diet high in trans fatty acids	Cardiovascular diseases	95.85	5.18
2019	Diet high in trans fatty acids	Cardiovascular diseases	92.92	5.48
2013	Diet low in calcium	Neoplasms	129.03	6.58
2019	Diet low in calcium	Neoplasms	128.71	7.05
2013	Diet low in fiber	Cardiovascular diseases	149.35	6.93
2013	Diet low in fiber	Neoplasms	17.62	0.89
2013	Diet low in fiber	Diabetes mellitus	19.84	0.18

2019	Diet low in fiber	Cardiovascular diseases	138.62	7.03
2019	Diet low in fiber	Neoplasms	17.23	0.94
2019	Diet low in fiber	Diabetes mellitus	22.00	0.18
2013	Diet low in fruits	Cardiovascular diseases	244.25	10.58
2013	Diet low in fruits	Neoplasms	124.35	6.52
2013	Diet low in fruits	Diabetes mellitus	48.16	0.40
2019	Diet low in fruits	Cardiovascular diseases	228.36	10.63
2019	Diet low in fruits	Neoplasms	112.73	6.31
2019	Diet low in fruits	Diabetes mellitus	51.85	0.40
2013	Diet low in legumes	Cardiovascular diseases	122.30	6.31
2019	Diet low in legumes	Cardiovascular diseases	110.52	6.36
2013	Diet low in milk	Neoplasms	161.61	8.15
2019	Diet low in milk	Neoplasms	165.80	8.97
2013	Diet low in nuts and seeds	Cardiovascular diseases	161.62	8.98
2013	Diet low in nuts and seeds	Diabetes mellitus	17.70	0.17
2019	Diet low in nuts and seeds	Cardiovascular diseases	155.87	9.50
2019	Diet low in nuts and seeds	Diabetes mellitus	20.31	0.18
2013	Diet low in polyunsaturated fatty acids	Cardiovascular diseases	49.95	2.64
2019	Diet low in polyunsaturated fatty acids	Cardiovascular diseases	47.60	2.74
2013	Diet low in seafood omega-3 fatty acids	Cardiovascular diseases	20.42	1.69
2019	Diet low in seafood omega-3 fatty acids	Cardiovascular diseases	48.25	3.49
2013	Diet low in vegetables	Cardiovascular diseases	40.07	2.05
2013	Diet low in vegetables	Neoplasms	5.54	0.29
2019	Diet low in vegetables	Cardiovascular diseases	38.66	2.21
2019	Diet low in vegetables	Neoplasms	5.43	0.30
2013	Diet low in whole grains	Cardiovascular diseases	324.65	16.71
2013	Diet low in whole grains	Neoplasms	145.78	7.34
2013	Diet low in whole grains	Diabetes mellitus	32.63	0.30
2019	Diet low in whole grains	Cardiovascular diseases	314.03	17.59
2019	Diet low in whole grains	Neoplasms	149.41	8.06
2019	Diet low in whole grains	Diabetes mellitus	37.34	0.31
2013	Drug use	Neoplasms	110.53	5.21
2013	Drug use	Other NCDs	215.28	3.99
2013	Drug use	Injurie	11.07	0.23
2019	Drug use	Neoplasms	115.82	5.78
2019	Drug use	Other NCDs	212.46	4.08
2019	Drug use	Injurie	9.49	0.20
2013	HPV infection	Neoplasms	NA	1.05
2019	HPV infection	Neoplasms	NA	1.31
2013	HTLV-1 infection	Neoplasms	NA	0.84
2019	HTLV-1 infection	Neoplasms	NA	0.79
2013	*Helicobacter pylori* infection	Neoplasms	NA	47.77
2019	*Helicobacter pylori* infection	Neoplasms	NA	51.05
2013	Hepatitis B virus infection	Neoplasms	87.80	3.90
2019	Hepatitis B virus infection	Neoplasms	82.95	3.87
2013	Hepatitis C virus infection	Neoplasms	426.59	23.96

2019	Hepatitis C virus infection	Neoplasms	418.91	25.13
2013	High LDL cholesterol	Cardiovascular diseases	1037.27	52.99
2019	High LDL cholesterol	Cardiovascular diseases	1013.70	57.18
2013	High body-mass index	Cardiovascular diseases	452.90	17.01
2013	High body-mass index	Neoplasms	198.29	9.83
2013	High body-mass index	Chronic kidney disease	99.08	3.89
2013	High body-mass index	Other NCDs	163.16	4.17
2013	High body-mass index	Chronic respiratory diseases	14.65	0.15
2013	High body-mass index	Diabetes mellitus	183.61	1.26
2019	High body-mass index	Cardiovascular diseases	439.78	17.37
2019	High body-mass index	Neoplasms	199.89	10.55
2019	High body-mass index	Chronic kidney disease	96.31	4.22
2019	High body-mass index	Other NCDs	178.78	5.42
2019	High body-mass index	Chronic respiratory diseases	14.87	0.16
2019	High body-mass index	Diabetes mellitus	209.82	1.28
2013	High fasting plasma glucose	Cardiovascular diseases	656.55	36.88
2013	High fasting plasma glucose	Neoplasms	232.13	13.56
2013	High fasting plasma glucose	Chronic kidney disease	262.88	12.11
2013	High fasting plasma glucose	Other NCDs	75.26	5.07
2013	High fasting plasma glucose	Diabetes mellitus	728.61	6.58
2019	High fasting plasma glucose	Cardiovascular diseases	743.98	44.34
2019	High fasting plasma glucose	Neoplasms	253.46	15.77
2019	High fasting plasma glucose	Chronic kidney disease	246.89	12.72
2019	High fasting plasma glucose	Other NCDs	99.23	7.04
2019	High fasting plasma glucose	Diabetes mellitus	829.51	6.98
2013	High systolic blood pressure	Cardiovascular diseases	2420.04	121.76
2013	High systolic blood pressure	Chronic kidney disease	395.29	19.94
2019	High systolic blood pressure	Cardiovascular diseases	2424.48	130.77
2019	High systolic blood pressure	Chronic kidney disease	413.69	23.25
2013	Intimate partner violence	Other NCDs	0.00	0.00
2013	Intimate partner violence	Injurie	0.00	0.00
2019	Intimate partner violence	Other NCDs	0.00	0.00
2019	Intimate partner violence	Injurie	0.00	0.00
2013	Kidney dysfunction	Cardiovascular diseases	420.29	23.91
2013	Kidney dysfunction	Chronic kidney disease	646.96	30.66
2013	Kidney dysfunction	Other NCDs	14.47	0.00
2019	Kidney dysfunction	Cardiovascular diseases	443.42	27.41
2019	Kidney dysfunction	Chronic kidney disease	649.69	34.28
2019	Kidney dysfunction	Other NCDs	17.29	0.00
2013	Low bone mineral density	Injurie	261.53	6.31
2019	Low bone mineral density	Injurie	298.31	7.38
2013	Low physical activity	Cardiovascular diseases	114.39	7.63
2013	Low physical activity	Neoplasms	46.06	2.77
2013	Low physical activity	Diabetes mellitus	53.60	0.60
2019	Low physical activity	Cardiovascular diseases	120.40	8.68
2019	Low physical activity	Neoplasms	49.92	3.20

2019	Low physical activity	Diabetes mellitus	63.22	0.67
2013	Tobacco smoking	Cardiovascular diseases	1345.30	55.45
2013	Tobacco smoking	Neoplasms	2673.38	144.97
2013	Tobacco smoking	Other NCDs	802.43	18.69
2013	Tobacco smoking	Chronic respiratory diseases	595.71	23.93
2013	Tobacco smoking	Diabetes mellitus	160.67	1.24
2013	Tobacco smoking	Injurie	33.41	0.72
2019	Tobacco smoking	Cardiovascular diseases	1167.19	49.56
2019	Tobacco smoking	Neoplasms	2430.99	140.31
2019	Tobacco smoking	Other NCDs	725.39	20.43
2019	Tobacco smoking	Chronic respiratory diseases	597.76	25.95
2019	Tobacco smoking	Diabetes mellitus	166.38	1.16
2019	Tobacco smoking	Injurie	31.88	0.68
2013	Unsafe sex	Neoplasms	0.00	0.00
2019	Unsafe sex	Neoplasms	0.00	0.00

* Data, except for hepatitis B and C virus infections, *Helicobacter pylori* infection, HPV infection, and HTLV-1 infection, were obtained from the GBD results tool: http://ghdx.healthdata.org/gbd-results-tool. DALYs: disability-adjusted life years; NCDs: non-communicable diseases; LDL: low-density lipoprotein; HPV: human papillomavirus; HTLV-1: human T-cell leukemia virus type 1. The order of the risk factors is sorted alphabetically.

Supplementary Table 8: Goals of Health Japan 21 (the second term) for prevention of onset and progression of non-communicable diseases

Target	Goal	Interim evaluation in 2018
Cancer		
• Reduction in age-adjusted mortality rate of cancer under age 75 (per 100,000)	73.9	A*
• Increase in rate of participation in cancer screenings	50% (40% for gastric, lung, and colorectal cancer)	A*
Cardiovascular disease		
• Reduction in age-adjusted mortality rate of cerebrovascular disease (CVD) and ischemic heart disease (IHD) (per 100,000)	CVD: Male 41.6, Female 24.7; IHD: Male 31.8, Female 13.7	A
• Improvement of hypertension (reduction in average systolic blood pressure)	Male 134 mmHg, Female 129 mmHg	A
• Reduction in percentage of adults with dyslipidemia	Those with total cholesterol over 240mg/dl: Male 10%, Female 17%; Those with LDL cholesterol over 160 mg/dl: Male 6.2%, Female 8.8%	B
• Reduction in number of definite and at-risk people with metabolic syndrome	25% less than 2008	B
• Increase in participation rates of specified health check-ups and specified health guidance	Specified health check-ups: 70%, Specified health guidance: 45%	A*
Diabetes		
• Reduction in complications (number of patients newly introduced to dialysis due to diabetic nephropathy)	15,000	B
• Increase in percentage of patients who continue treatment	75%	B
• Decrease in percentage of individuals with elevated blood glucose levels (HbA1c (NGSP)≧8.4%)	1.0%	A
• Prevent increase in number of diabetic persons	10,000,000	B
• Reduction in number of definite and at-risk people with metabolic syndrome	25 less than 2008	B
• Increase in participation rates of specified health check-ups and health guidance	Specified health check-ups: 70%, Specified health guidance: 45%	A*
COPD		
• Increase recognition of COPD	80%	B

A: Improving; A*: Improving, but if the current trend continues, it is unlikely that the goal will be reached by the final evaluation; B: No change. Data were adapted from the interim evaluation report of the Health Japan 21 progress in 2018 (Ministry of Health, Labour and Welfare. Report on the interim evaluation of Health Japan 21 (2nd term) [Japanese]. 2018. https://www.mhlw.go.jp/content/000378318.pdf (accessed December 14, 2021)).

Supplementary Table 9: Goals of Health Japan 21 (the second term) for improvement of lifestyle

Target	Goal	Interim evaluation in 2018
Nutrition and dietary habits		
• Increase in percentage of individuals maintaining ideal body weight (Reduction in percentage of obese individuals [BMI 25 and more] and underweight individuals [BMI less than 18.5])	Obese males in their 20s to 60s: 28%; Obese females in their 40s to 60s: 19%; Underweight females in their 20s: 20%	B
• Increase in percentage of individuals who consume appropriate quality and quantity of food		B
o Increase in percentage of individuals who eat a balanced diet with staple food, main dish and side dish more than twice a day	80%	
o Decrease in mean salt intake	8g	
o Increase in consumption of vegetables and fruits	Mean daily intake of vegetables: 350g; Individuals who consume fruit less than 100g per day: 30%	
• Increase in dining with family regularly (decrease in percentage of children who eat alone)	To decrease	B
• Increase in number of corporations in food industry that supply food product low in salt and fat	Registered corporations: 100; Registered restaurants 30,000 locations	A
• Increase in percentage of specific foodservice facilities that plan, cook, and evaluate and improve nutritional content of menu based on the needs of clients	80%	A*
Physical activity and exercise		
• Increase in daily number of steps	20–64 years old Male 9,000 steps, Female 8,500 steps; Over 65 years old Male 7,000 steps, Female 6,000 steps	B
• Increase in percentage of individuals who regularly exercise	20-64 years old Male 36%, Female 33%; Over 65 years old Male 58% Female 48%	B
• Increase in number of local governments that offer community development and environment to promote physical activity	47 prefectures	A
Rest		
• Reduction in percentage of individuals who do not take rest through sufficient sleep	15%	B
• Reduction in percentage of employees who work 60 hours or more per week	5.0%	A*
Alcohol drinking		
• Reduction in percentage of individuals who consume alcohol over recommended limits (male > 40 g, female > 20 g per day)	Male 13%, Female 6.4%	B
• Eradication of underage drinking	0%	A
• Eradication of alcohol consumption among pregnant women	0%	A*
Tobacco smoking		
• Reduction in percentage of adult smoking rate	12%	A*

• Eradication of underage smoking	0%	A
• Eradication of smoking during pregnancy	0%	A*
• Reduction in percentage of individuals who are exposed to passive smoking at home, workplace, restaurants, governmental institutions, and medical institutions	Governmental institutions 0%; Medical institutions 0%; Workplace 0%; Home 3%; Restaurants 15%	A*
Dental and oral health		
• Maintenance and improvement of oral function (increase in percentage of individuals in their 60s with good mastication)	80%	B
• Prevention of tooth loss		A
○ Increase in percentage of 80-year-old individuals with over 20 teeth remaining	50%	
○ Increase in percentage of 60-year-old individuals with over 24 teeth remaining	70%	
○ Increase in percentage of 40-year-old individuals with all teeth remaining	75%	
• Decrease in percentage of individuals with periodontal disease		C
○ Decrease in percentage of individuals in 20s with gingivitis	25%	
○ Decrease in percentage of individuals in 40s with progressive periodontitis	25%	
○ Decrease in percentage of individuals in 60s with progressive periodontitis	45%	
• Increase in number of children without dental caries		A
○ Increase in number of prefectures where over 80% of 3-year-old children have no dental caries	23 prefectures	
○ Increase in number of prefectures where 12-year-old children have less than 1 mean decayed, missing, and filled teeth (dmft)	28 prefectures	
• Increase in percentage of individuals who participated in dental check-up	28 prefectures	A

A: Improving; A*: Improving, but if the current trend continues, it is unlikely that the goal will be reached by the final evaluation; B: No change. Data were adapted from the interim evaluation report of the Health Japan 21 progress in 2018 (Ministry of Health, Labour and Welfare. Report on the interim evaluation of Health Japan 21 (2nd term) [Japanese]. 2018. https://www.mhlw.go.jp/content/000378318.pdf (accessed December 14, 2021)).
